# Glomerular Expression of S100A8 in Lupus Nephritis: An Integrated Bioinformatics Analysis

**DOI:** 10.3389/fimmu.2022.843576

**Published:** 2022-04-27

**Authors:** Wei Qijiao, Chen Zhihan, Panashe Makota, Yan Qing, Gao Fei, Wang Zhihong, Lin He

**Affiliations:** ^1^Fujian Provincial Hospital, Fuzhou, China; ^2^Fujian Medical University Provincial Clinical Medical College, Fuzhou, China

**Keywords:** lupus nephritis, differentially expressed genes, microarray, immunofluorescence, S100A8

## Abstract

**Introduction:**

Lupus nephritis (LN) is a major risk factor of morbidity and mortality. Glomerular injury is associated with different pathogeneses and clinical presentations in LN patients. However, the molecular mechanisms involved are not well understood. This study aimed to explore the molecular characteristics and mechanisms of this disease using bioinformatics analysis.

**Methods:**

To characterize glomeruli in LN, microarray datasets GSE113342 and GSE32591 were downloaded from the Gene Expression Omnibus database and analyzed to determine the differentially expressed genes (DEGs) between LN glomeruli and normal glomeruli. Functional enrichment analyses and protein–protein interaction network analyses were then performed. Module analysis was performed using the Search Tool for the Retrieval of Interacting Genes/Proteins and Cytoscape software. Immunofluorescence staining was performed to identify the glomerular expression of S100A8 in various International Society of Nephrology/Renal Pathology Society (ISN/RPS) class LN patients. The image of each glomerulus was acquired using a digital imaging system, and the green fluorescence intensity was quantified using Image-Pro Plus software.

**Results:**

A total of 13 DEGs, consisting of 12 downregulated genes and one upregulated gene (S100A8), were identified in the microarray datasets. The functions and pathways associated with the DEGs mainly include inflammatory response, innate immune response, neutrophil chemotaxis, leukocyte migration, cell adhesion, cell–cell signaling, and infection. We also found that monocytes and activated natural killer cells were upregulated in both GSE113342 and GSE32591. Glomerular S100A8 staining was significantly enhanced compared to that in the controls, especially in class IV.

**Conclusions:**

The DEGs identified in the present study help us understand the underlying molecular mechanisms of LN. Our results show that glomerular S100A8 expression varies in different pathological types; however, further research is required to confirm the role of S100A8 in LN.

## Biographical Note

We use a bioinformatics method to obtain the DEGs between LN glomerulus and normal glomerulus and performed immunofluorescence staining to identify the expression of S100A8 in various ISN/RPS class LN patients.

## Significance and Innovations

➣Monocytes and activated NK cells were upregulated in LN glomerulus.
➣Glomerular S100A8 is different in different pathological types.
➣The glomerular S100A8 staining was obviously enhanced compared with the controls, especially in class IV.

## Introduction

Systemic lupus erythematosus (SLE) is an autoimmune disease characterized by immune inflammation, which can affect multiple organs. It is known to affect the kidneys in approximately 50% of patients (1). Lupus nephritis (LN) is a major risk factor of morbidity and mortality. It was found that glomerular injury, including that of mesangial cells, endothelial cells, and podocytes, is associated with different pathogeneses and clinical presentations in LN patients (2). Intense efforts have been made to elucidate the pathogenesis and molecular mechanisms of LN; however, they are still not well understood (3). Therefore, it is necessary to further explore the molecular characteristics and mechanisms of the disease. To characterize glomeruli in LN using bioinformatics analysis, microarray datasets GSE113342 and GSE32591 were downloaded from the Gene Expression Omnibus (GEO) database and analyzed to determine the differentially expressed genes (DEGs) between the LN glomerulus and normal glomerulus. Functional enrichment analyses and protein–protein interaction (PPI) network analyses were then performed. Module analysis was performed using the Search Tool for the Retrieval of Interacting Genes/Proteins (STRING) and Cytoscape software.

S100A8 was identified as one of the 13 DEGs. However, its mRNA expression was different in the abovementioned two microarray datasets. S100A8 (also known as MRP8) is a calcium-binding protein belonging to the S100 family (4). It is mainly expressed in granulocytes and mononuclear blood cells, such as neutrophils and macrophages (5). S100A8 is expressed in various autoimmune diseases, such as systemic sclerosis, psoriasis, dermatomyositis, and Sjogren’s syndrome (6). Some studies have also found that serum and urine S100A8 levels are elevated in patients with LN (7, 8). To our knowledge, the glomerular expression of S100A8 in various ISN/RPS class LN patients is unknown. In this study, immunofluorescence staining and semiquantitative analysis were performed, and the relationship between glomerular expression of S100A8 and clinical data, such as disease activity or urinary protein measurements, was explored.

## Methods

### Microarray Data

NCBI GEO (http://www.ncbi.nlm.nih.gov/geo) (9) is a public functional genomics data repository with high-throughput gene expression data, chips, and microarrays. We used “lupus nephritis” (keywords) to search, and there were 335 results in the GEO Database. Then, we selected *Homo sapiens*, and 301 results were left. We browsed 301 links in detail. In this study, we want to explore the DEGs between LN glomerulus and normal glomerulus. In the first step, we selected LN kidney samples. And only 5 datasets were left. They were GSE112943, GSE127797, GSE113342, GSE32591, and GSE69438. Then, only glomeruli and tubulointerstitium separated samples were what we needed, and GSE113342 and GSE32591 were selected. The two gene expression datasets GSE113342 (10) and GSE32591 (11) were downloaded. The GSE113342 dataset contained 14 LN glomerulus biopsy samples and six normal glomerulus biopsy samples. GSE32591 contained 32 LN glomerulus biopsy samples and 14 normal glomerulus biopsy samples.

### Identification of Differentially Expressed Genes

The GEO2R online tool was used to identify DEGs between LN and normal glomerular biopsy samples. Log fold change (FC) >1 and P-value <0.01 were considered statistically significant. The raw data were checked online using Venn software to detect common DEGs. DEGs with log FC <0 were considered downregulated, whereas DEGs with log FC >0 were considered upregulated.

### Functional Enrichment and Protein–Protein Interaction Analysis

The Database for Annotation, Visualization, and Integrated Discovery (DAVID; http://david.ncifcrf.gov) (12) is an online biological information database. The Kyoto Encyclopedia of Genes and Genomes (KEGG) is a database resource for understanding high-level functions and biological systems from large-scale molecular datasets generated using high-throughput experimental technologies (13). Gene Ontology (GO) is a major bioinformatics tool for annotating genes and analyzing their biological processes (14). DAVID 6.8 Bioinformatics Resources was used for pathway annotations. Statistical significance was set at P < 0.05. A PPI network was established using the STRING (version 10.0) (15) online search tool, and an interaction with a combined score >0.4 was considered statistically significant. The PPI network was visualized using Cytoscape (version 3.7.2).

### Profiling Infiltrating Immune Cells With CIBERSORT in the Glomeruli

To assess the expression changes in immune cells and obtain the proportion of various types of immune cells from the glomerulus, we used the online CIBERSORT algorithm (https://cibersort.stanford.edu/). The GSE113342 and GSE32591 series matrix txt format files were downloaded from the NCBI GEO website, and the glomerular expression data were selected (GSM3103966, GSM3103968, GSM3103970, GSM3103972, GSM3103974, GSM3103976, GSM3103978, GSM3103980, GSM3103982, GSM3103984, GSM3103986, GSM3103988, GSM3103990, GSM3103992, GSM3103994-99, GSM807889-7934). Differences in 22 immune cells between normal and lupus glomeruli were analyzed.

### Patients

All protocols were approved by the ethics committee of the Fujian Provincial Hospital. Overall, 30 LN patients with a mean age of 32.70 ± 12.17 years were included. Six types of pathological classifications (Classes II, III, IV, V, III+V, and IV+V) were used, with five patients in each pathological classification. Normal renal tissues from 5 patients with a mean age of 55.47 ± 8.82 years who underwent nephrectomy due to renal tumors were used as normal controls.

### Immunofluorescence Staining of Glomerular S100A8

Renal biopsy specimens were embedded in an OCT mixture (Sakura, Hayward, CA, USA) and sliced into 5-μm frozen sections (16). The mouse anti-S100A8 antibody (Proteintech Group, Inc., Chicago, IL, USA) was used. Rabbit anti-synaptopodin antibody (Proteintech Group, Inc., Chicago, IL, USA) was used as a podocyte marker for double immunofluorescence staining. Goat anti-rabbit IgG/Alexa Fluor 594 antibody and goat anti-mouse IgG/Alexa Fluor 488 antibody (BIOGOT, Nanjing, China) were used to visualize the different proteins. 4',6-diamidino-2-phenylindole (DAPI) was used to stain the nucleus. Images were captured using a fluorescence microscope (Nikon Eclipse C1, Japan). Integral optical density (IOD) and the area ratio (AR) of the positively stained area to the glomerular area were used as semiquantitative values of the expression of S100A8. We used Image-Pro Plus software (17).

### Correlation of Glomerular Expression of S100A8 With Clinical and Laboratory Data

The clinical data are from the cohort used for fluorescence staining of S100A8 in our study. Thirty LN patients were used for this correlation analysis. Six types of pathological classifications (Classes II, III, IV, V, III+V, and IV+V) were used, with five patients in each pathological classification.

### Statistical Analysis

SPSS (version 21.0; SPSS Inc., Chicago, IL, USA) was used to compare the differences between the two groups using the *t*-test or Mann–Whitney U test. The ratio was calculated using the χ^2^ test. Spearman correlation analysis was performed between clinical data and glomerular S100A8 expression levels. Statistical significance was set at P < 0.05.

## Results

### Differentially Expressed Gene Identification

**Figure 1:**
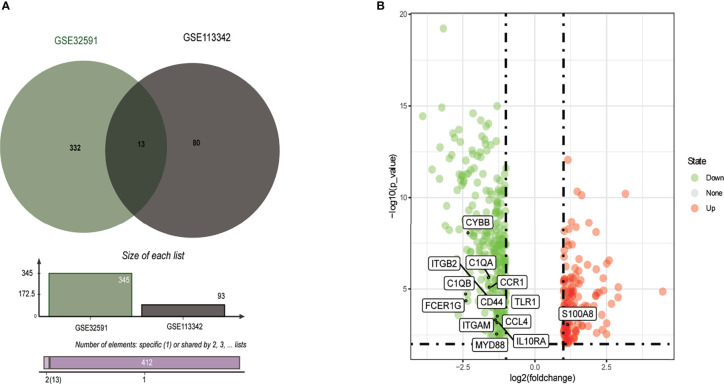
Identification of common DEGs from GSE32591 and GSE113342 datasets. **(A)** Venn diagram of DEGs based on the two GEO datasets. **(B)** Volcano plot of the 13 DEGs. Red, upregulation; green, downregulation. DEGs, differentially expressed genes; GEO, Gene Expression Omnibus.

From the GSE113342 dataset, 93 DEGs were successfully identified, including 53 upregulated and 40 downregulated genes. From the GSE32591 dataset, 345 DEGs, involving 97 upregulated and 248 downregulated genes, were observed. Out of all the DEGs, 13 were common between the two datasets, as shown in the Venn diagram (**Figure 1A**). These 13 DEGs discovered between the LN glomerulus and the normal glomerulus based on the two microarray datasets consisted of 12 downregulated genes and one upregulated gene (S100A8). The 13 DEGs are plotted in **Figure 1B**, where the red and green dots represent upregulated and downregulated genes, respectively.

### Gene Ontology Annotation and Kyoto Encyclopedia of Genes and Genomes Pathway Enrichment Analyses

**Figure 2:**
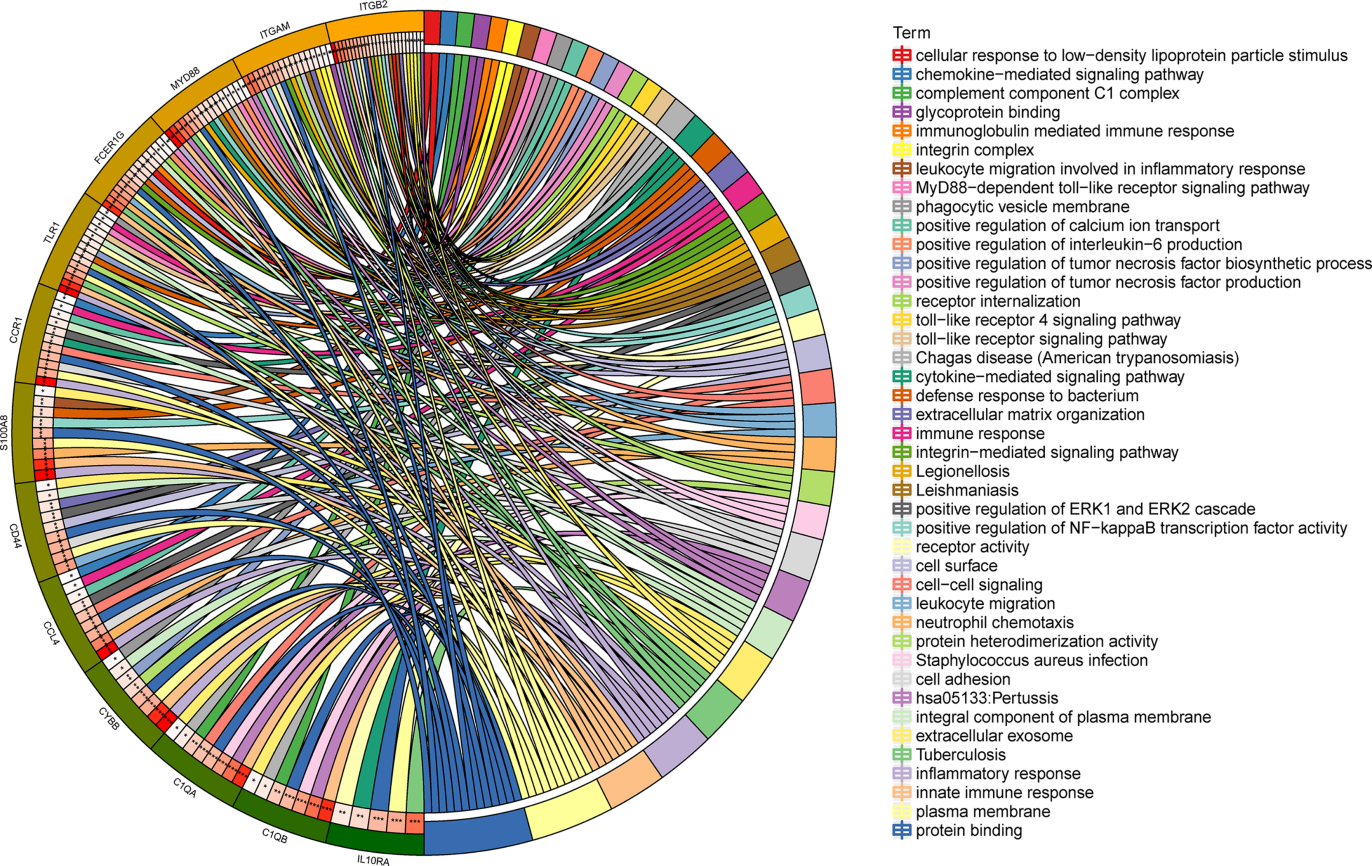
Distribution of integrated DEGs in LN glomerulus for different enriched functions. The DEG enrichment of BP, MF, CC, and KEGG pathways (P < 0.05).

To gain deeper insight into the biological roles of these 13 DEGs, functional and pathway enrichment analyses were performed using DAVID. The enriched GO terms and KEGG pathways are shown in **Table 1** and **Figure 2**. KEGG pathway analysis revealed that the DEGs were mainly associated with infections and the Toll-like receptor (TLR) signaling pathway. GO biological process analysis indicated that the 13 DEGs were significantly associated with inflammatory response, innate immune response, neutrophil chemotaxis, leukocyte migration, cell adhesion, and cell–cell signaling. The top 3 significantly enriched terms regarding changes in cell component of DEGs were plasma membrane, extracellular exosome, and integral component of the plasma membrane. Changes in molecular function are primarily associated with protein binding.

**Table 1:** GO and KEGG pathway enrichment analysis of DEGs in LN glomerulus.

	Term	Description	Count in gene set	P-value
KEGG_PATHWAY	hsa05133	Pertussis	5	4.06E-06
	hsa05152	Tuberculosis	6	4.34E-06
	hsa05150	*Staphylococcus aureus* infection	4	7.22E-05
	hsa05134	Legionellosis	3	0.003179
	hsa05140	Leishmaniasis	3	0.00544
	hsa05142	Chagas disease (American trypanosomiasis)	3	0.011392
	hsa04620	Toll-like receptor signaling pathway	3	0.011815
Biological processes (BP)	GO:0006954	Inflammatory response	7	1.05E-07
	GO:0045087	Innate immune response	7	2.21E-07
	GO:0030593	Neutrophil chemotaxis	4	1.24E-05
	GO:0050900	Leukocyte migration	4	7.85E-05
	GO:0007155	Cell adhesion	5	2.29E-04
	GO:0007267	Cell–cell signaling	4	6.80E-04
	GO:0007229	Integrin-mediated signaling pathway	3	0.002185
	GO:0019221	Cytokine-mediated signaling pathway	3	0.003787
	GO:0051092	Positive regulation of NF-kappaB transcription factor activity	3	0.003901
	GO:0042742	Defense response to bacterium	3	0.004618
	GO:0071404	Cellular response to low-density lipoprotein particle stimulus	2	0.006415
	GO:0070374	Positive regulation of ERK1 and ERK2 cascade	3	0.006655
	GO:0002523	Leukocyte migration involved in the inflammatory response	2	0.007835
	GO:0016064	Immunoglobulin-mediated immune response	2	0.007835
	GO:0042535	Positive regulation of tumor necrosis factor biosynthetic process	2	0.007835
	GO:0030198	Extracellular matrix organization	3	0.008283
	GO:0034142	Toll-like receptor 4 signaling pathway	2	0.012792
	GO:0051928	Positive regulation of calcium ion transport	2	0.018429
	GO:0002224	Toll-like receptor signaling pathway	2	0.019131
	GO:0002755	MyD88-dependent Toll-like receptor signaling pathway	2	0.023337
	GO:0031623	Receptor internalization	2	0.03031
	GO:0032755	Positive regulation of interleukin-6 production	2	0.031699
	GO:0032760	Positive regulation of tumor necrosis factor production	2	0.033086
	GO:0006955	Immune response	3	0.035053
	GO:0070098	Chemokine-mediated signaling pathway	2	0.049591
Cell component (CC)	GO:0005886	Plasma membrane	10	1.71E-04
	GO:0005602	Complement component C1 complex	2	0.001317
	GO:0009986	Cell surface	4	0.00471
	GO:0005887	Integral component of plasma membrane	5	0.010799
	GO:0008305	Integrin complex	2	0.01764
	GO:0070062	Extracellular exosome	6	0.026716
	GO:0030670	Phagocytic vesicle membrane	2	0.038177
Molecular function (MF)	GO:0005515	Protein binding	13	3.93E-04
	GO:0046982	Protein heterodimerization activity	4	0.003795
	GO:0004872	Receptor activity	3	0.009973
	GO:0001948	Glycoprotein binding	2	0.045254

GO, Gene Ontology; KEGG, Kyoto Encyclopedia of Genes and Genomes; DEGs, differentially expressed genes; LN, lupus nephritis; ERK, extracellular regulated protein kinases.

### Protein–Protein Interaction and Modular Analysis

**Figure 3:**
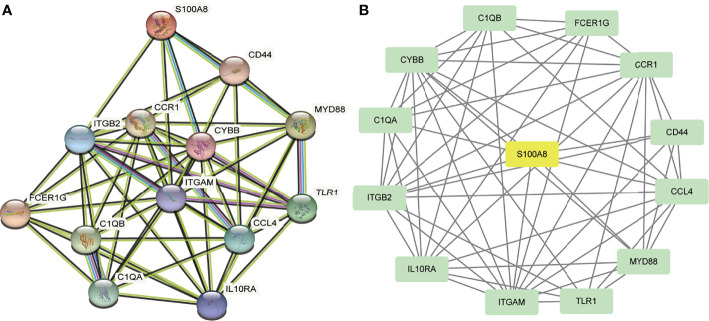
PPI network and the significant module of DEGs. **(A)** The PPI network of DEGs. **(B)** The significant module was obtained from PPI network constructed using Cytoscape with 13 nodes and 57 edges. S100A8 is marked in yellow, and downregulated genes are marked in green.

A total of 13 DEGs were imported into the PPI network complex, comprising 13 nodes and 57 edges, including 12 downregulated genes and the upregulated gene S100A8 (**Figure 3**). We then applied Cytotype MCODE for further analysis, and the results are shown in **Figure 3B**.

### Infiltrating Immune Cells in the Glomeruli

**Figure 4:**
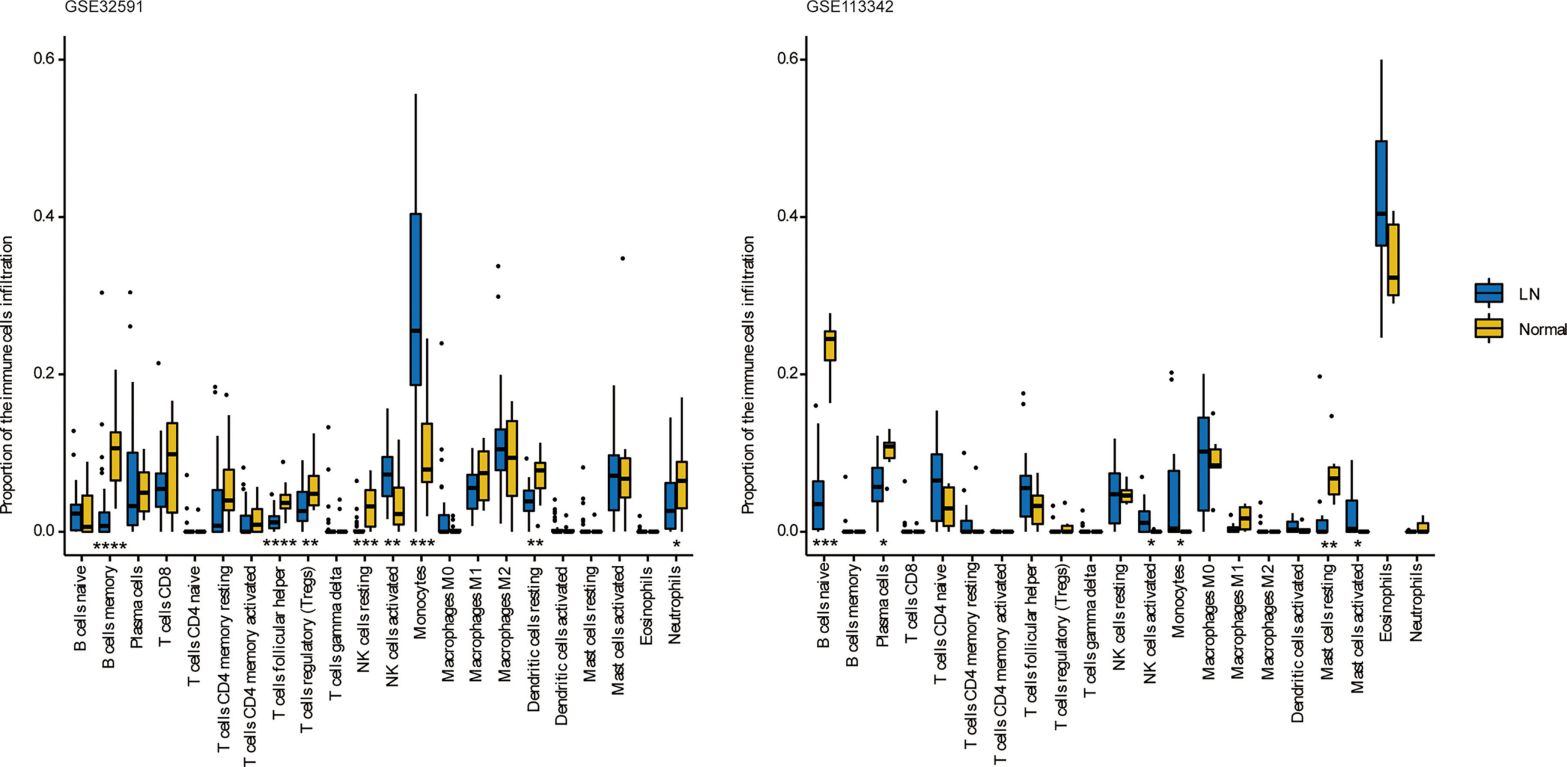
The differences of 22 immune cells between normal and lupus glomeruli. Monocytes and activated NK cells were upregulated in GSE32591 and GSE113342. *P < 0.05; **P < 0.01; ***P < 0.001; ****P < 0.0001.

**Figure 5:**
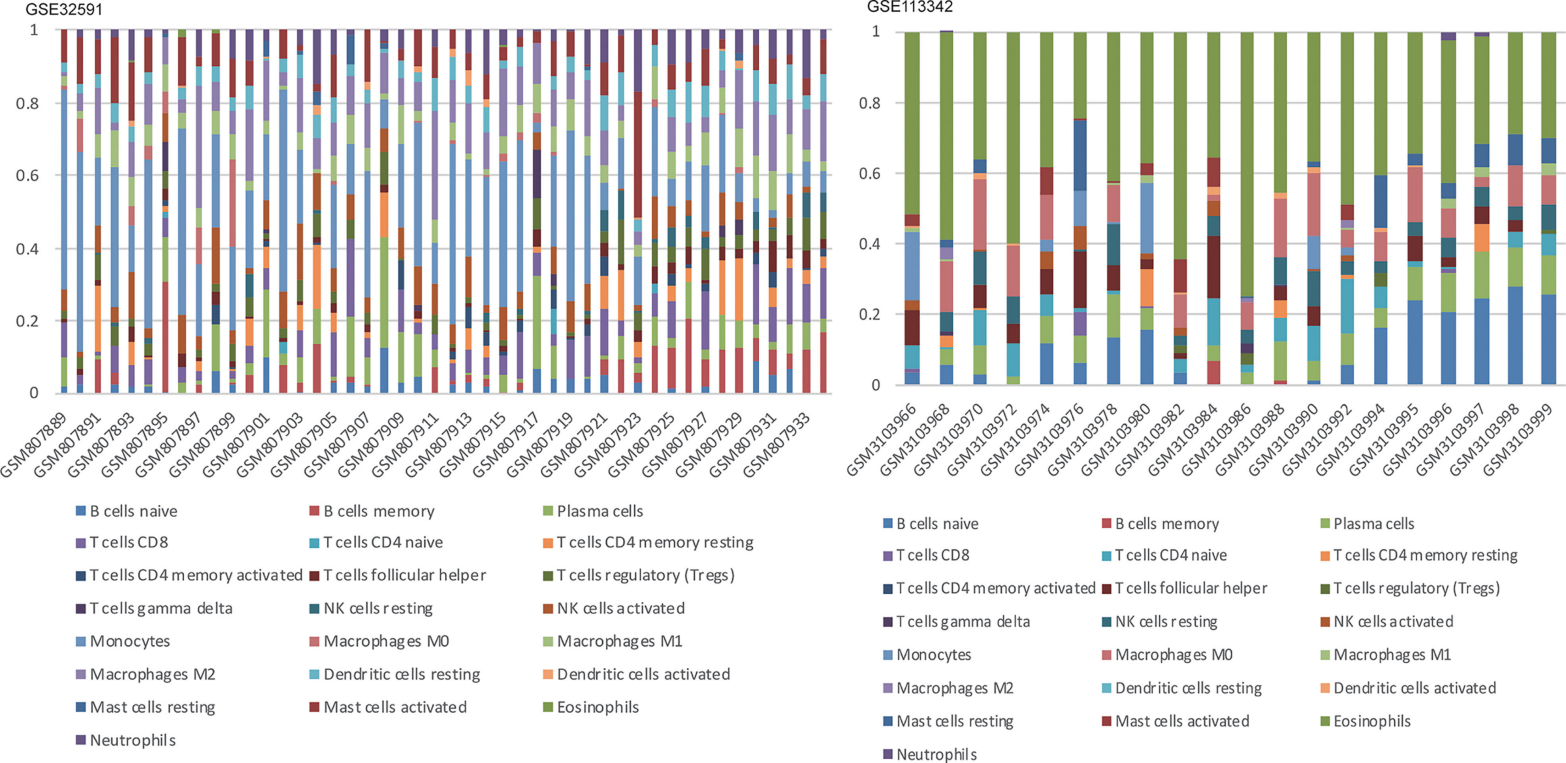
Stacked bar charts of 22 immune cell proportions in the glomeruli. In GSE32591, monocytes accounted for the majority of all infiltrating cells in the glomeruli. While in GSE113342, eosinophils are the majority.

We can conclude that monocytes (GSE32591) and eosinophils (GSE113342) accounted for the majority of all infiltrating cells in the glomerulus, especially in patients with LN. The differential expression proportion of immune-infiltrating cells in the LN and normal groups is shown in **Figures 4**, **5**. Based on analyses of the GSE32591 database, we found that memory B cells, follicular helper T cells, regulatory T cells (Tregs), and resting natural killer (NK) cells were downregulated in LN glomeruli. In addition, monocytes and activated NK cells were upregulated. Analyses of the GSE113342 database revealed that naive B cells, plasma cells, and resting mast cells were downregulated, and monocytes and activated NK cells were upregulated, similar to the observations from GSE32591. In addition, activated mast cells and eosinophils were also upregulated.

### Clinical and Laboratory Information of the Lupus Nephritis Patients

No significant age- or sex-dependent differences were found among the renal biopsies of different LN groups. There were some differences in Systemic Lupus Erythematosus Disease Activity Index (SLEDAI), Creatinine (Cr), Blood Urea Nitrogen (BUN), C3, C4, and Albumin (Alb) among various ISN/RPS class LN patients. In patients with LN, no significant difference in 24-h urinary protein measurements was found. The details are presented in **Table 2**.

**Table 2:** Clinical and laboratory information of the LN patients.

Group	Control	LN patients	Class II	Class III	Class IV	Class V	Class III+V	Class IV+V	P-value (Control vs. LN)	P-value (Among LN)
Age (years)	55.47 ± 8.82	32.70 ± 12.17	31.60 ± 11.89	36.9 ± 17.61	31.00 ± 12.75	35.40 ± 11.15	33.40 ± 12.08	30.80 ± 11.97	<0.01	0.05
Sex (F/M)	10/5	23/7	3/2	3/2	4/1	5/0	3/2	5/0	0.49	0.44
SLEDAI	NA	18.57 ± 4.44	14.40 ± 2.97	15.8 ± 2.68	20.4 ± 2.97	17.2 ± 4.15	22.4 ± 3.36	21.2 ± 5.02	NA	0.009
Cr (μmol/L)	75.27 ± 21.30	75.50 ± 72.43	52.00 ± 10.89	64.00 ± 10.08	69.80 ± 10.46	134.20 ± 175.51	56.00 ± 14.25	77.00 ± 31.96	0.99	0.04
BUN (mmol/L)	4.89 ± 0.86	7.00 ± 4.83	5.38 ± 1.53	7.20 ± 1.60	4.92 ± 0.64	8.70 ± 8.47	3.88 ± 0.90	11.90 ± 6.04	0.03	<0.01
24-h UTP or Urine protein (g/24 h)	All negative	1.66 ± 1.71	0.95 ± 1.10	0.70 ± 0.47	1.53 ± 0.65	2.18 ± 1.57	2.16 ± 1.75	2.40 ± 3.25	<0.01	0.54
C3 (g/L)	NA	0.52 ± 0.09	0.53 ± 0.24	0.68 ± 0.37	0.29 ± 0.14	0.89 ± 0.35	0.36 ± 0.16	0.37 ± 0.09	NA	0.007
C4 (g/L)	NA	0.09 ± 0.07	0.08 ± 0.04	0.11 ± 0.08	0.07 ± 0.05	0.18 ± 0.13	0.05 ± 0.03	0.06 ± 0.03	NA	0.06
Alb (g/L)	NA	32.33 ± 6.58	38.20 ± 4.15	33.20 ± 7.40	26.60 ± 7.40	35.80 ± 3.70	31.60 ± 2.61	28.60 ± 6.95	NA	0.04
Glomerular S100A8-AR	0.002 (0.001, 0.005)	0.010 (0.002, 0.028)	0.002 (0.001, 0.005)	0.026 (0.020, 0.075)	0.059 (0.035, 0.107)	0.003 (0.000, 0.003)	0.013 (0.006, 0.033)	0.018 (0.008, 0.034)	0.002	<0.01
Glomerular S100A8-IOD	5.967 (2.149, 16.933)	24.805 (5.647, 87.4068)	6.982 (2.161, 13.752)	82.603 (20.480, 147.444)	227.417 (133.910, 407.012)	1.027 (0.000, 10.880)	39.839 (14.546, 90.071)	62.562 (20.069, 100.833)	0.006	<0.01

### Glomerular Expression of S100A8 in Various ISN/RPS Class Lupus Nephritis Patients

**Figure 6:**
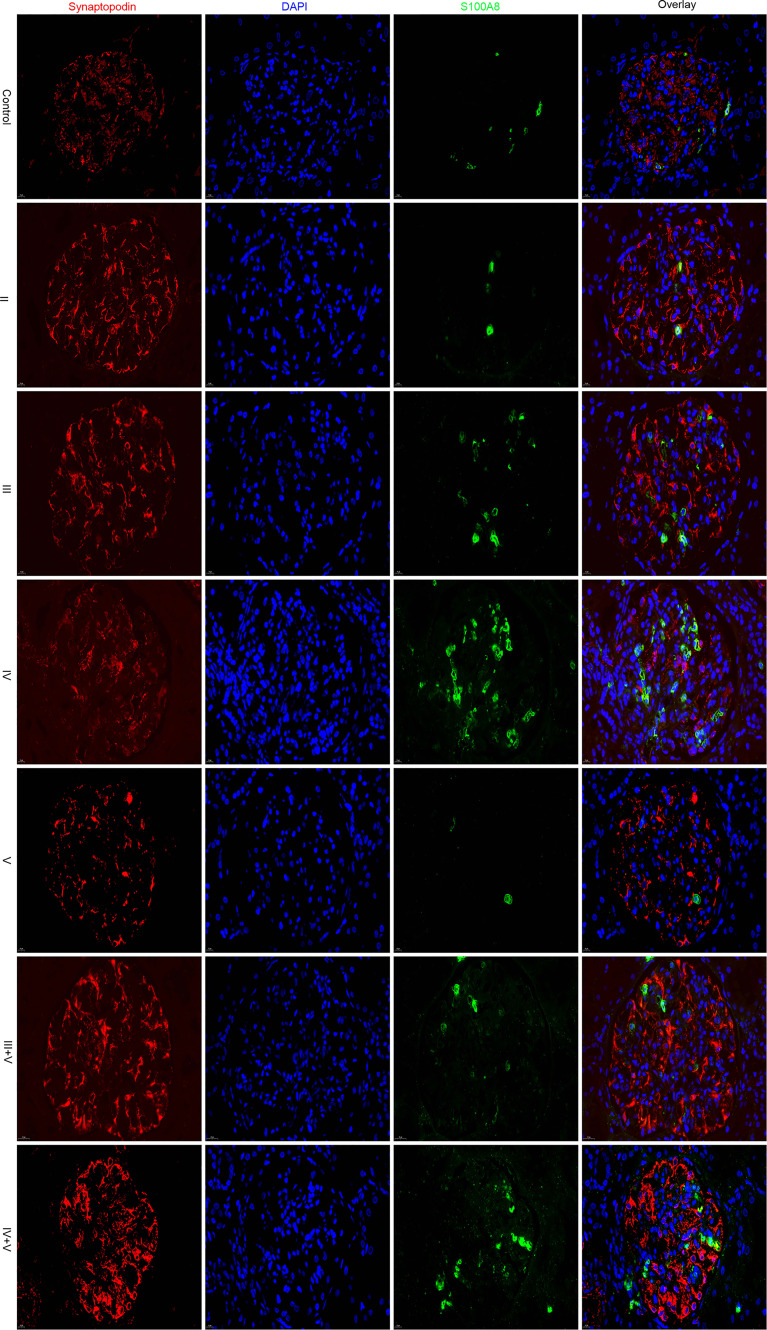
Glomerular expression of S100A8 in various ISN/RPS class LN patients.

Using immunofluorescence microscopy, the glomerular staining of S100A8 in the controls was found to be weak, and the staining in patients with classes II and V was similar to that of the controls. Glomerular staining was markedly enhanced in Class IV. We found the S100A8 proteins to be distributed throughout the glomerulus, and S100A8 did not colocalize with the podocyte marker synaptopodin (**Figure 6**).

We conducted a semiquantitative analysis and found a significant increase in IOD and AR in LN compared with that of the controls. However, no significant differences were found in the glomerular expression of S100A8 between the control and class II groups or control and class V groups (**Tables 2**, **3**).

**Table 3:** Glomerular expression of S100A8 in various ISN/RPS class LN patients.

Glomerular S100A8-AR/IOD	Control	LN patients	Class II	Class III	Class IV	Class V	Class III+V	Class IV+V
P value								
Control	0.002 (0.001, 0.005)/5.967 (2.149, 16.933)	0.002/0.006	0.955/0.966	<0.001/0.001	<0.001/<0.001	0.856/0.916	0.002/0.013	<0.001/<0.001
LN patients	0.002/0.006	0.010 (0.002, 0.028)/24.805 (5.647, 87.4068)	<0.001/<0.001	0.003/0.387	<0.001/<0.001	0.001/0.005	0.988/0.693	0.601/0.312
Class II	0.955/0.966	<0.001/<0.001	0.002 (0.001, 0.005)/6.982 (2.161, 13.752)	<0.001/0.001	<0.001/<0.001	0.814/0.884	<0.001/0.015	<0.001/<0.001
Class III	<0.001/0.001	0.003/0.387	<0.001/0.001	0.026 (0.020, 0.075)/82.603 (20.480, 147.444)	<0.001/<0.001	<0.001/0.001	<0.001/0.218	<0.001/0.887
Class IV	<0.001/<0.001	<0.001/<0.001	<0.001/<0.001	<0.001/<0.001	0.059 (0.035, 0.107)/227.417 (133.910, 407.012)	<0.001/<0.001	<0.001/<0.001	<0.001/<0.001
Class V	0.856/0.916	0.001/0.005	0.814/0.884	<0.001/0.001	<0.001/<0.001	0.003 (0.000, 0.003)/1.027 (0.000, 10.880)	<0.001/0.016	<0.001/<0.001
Class III+V	0.002/0.013	0.988/0.693	<0.001/0.015	<0.001/0.218	<0.001/<0.001	<0.001/0.016	0.013 (0.006, 0.033)/39.839 (14.546, 90.071)	0.632/0.208
Class IV+V	<0.001/<0.001	0.601/0.312	<0.001/<0.001	<0.001/0.887	<0.001/<0.001	<0.001/<0.001	0.632/0.208	0.018 (0.008, 0.034)/62.562 (20.069, 100.833)

### Correlation of Glomerular Expression of S100A8 With Clinical and Laboratory Data

**Figure 7:**
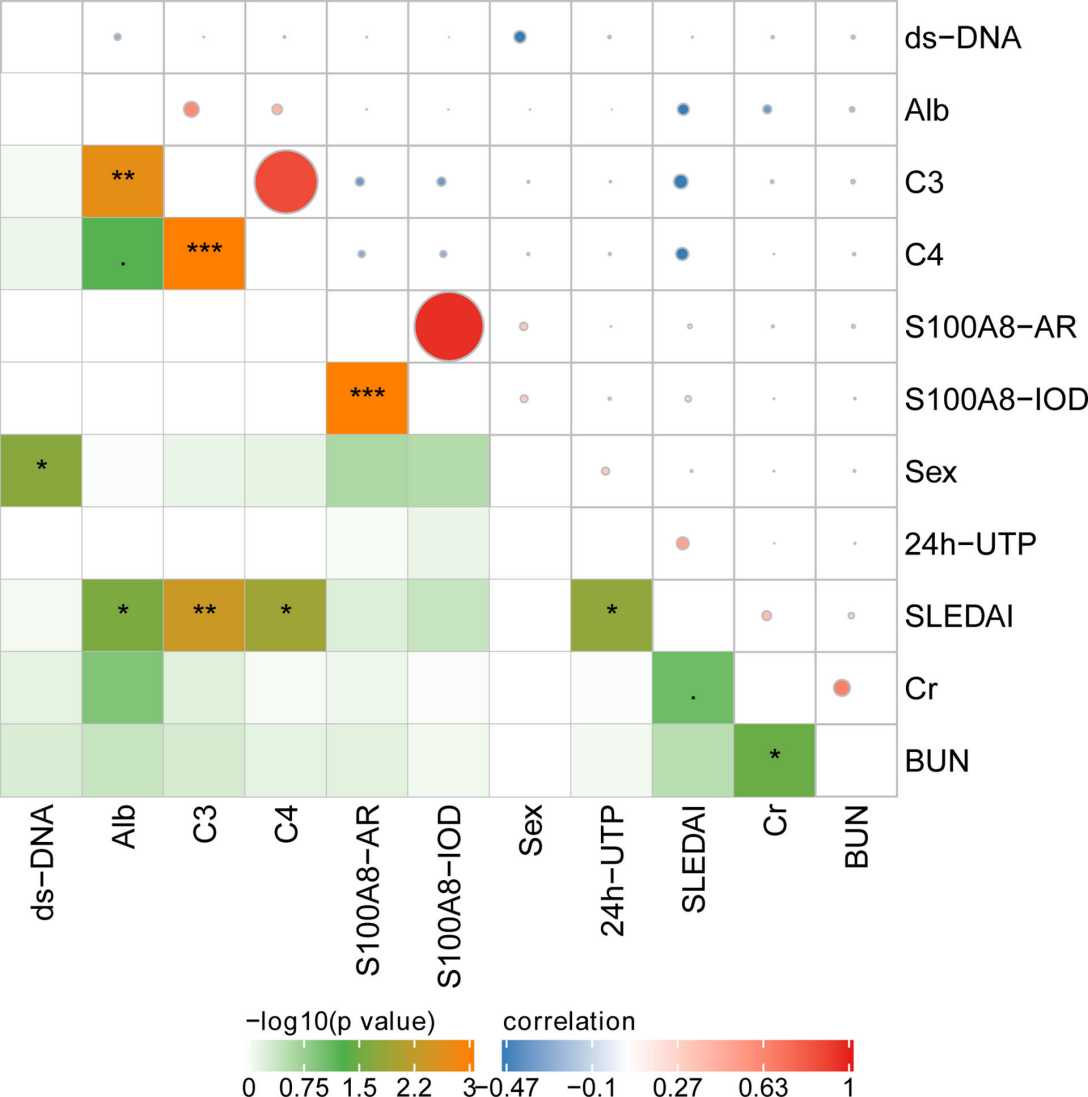
The correlation of glomerular expression of S100A8 with clinical and laboratory data. *P < 0.05; **P < 0.01;***P < 0.001.

The IOD of S100A8 positively correlated with the AR of S100A8. However, the IOD and AR of S100A8 did not correlate with clinical and laboratory data. Correlations were observed among anti-ds DNA, SLEDAI, Cr, BUN, C3, C4, Alb, and 24-h urinary total protein (UTP) in various ISN/RPS class LN patients. The details are presented in **Figure 7**.

## Discussion

In this study, we used bioinformatics analysis to identify 13 DEGs (CYBB, C1QA, C1QB, ITGB2, ITGAM, IL10RA, TLR1, MYD88, CCL4, CD44, CCR1, FCER1G, and S100A8) that were common between LN and normal glomeruli based on gene expression profiles obtained from the GSE113342 and GSE32591 datasets.

Proteins expressed by these genes are distributed in a variety of inflammatory cells. They are also chemokine receptors for inflammatory cells (18–28). Through enrichment analysis, we found that these genes were mainly related to the inflammatory response, innate immune response, neutrophil chemotaxis, leukocyte migration, cell adhesion, and cell–cell signaling. These factors are closely related to the pathogenesis of LN.

We also observed differential expression of immune cells, including T cells, B cells, NK cells, and macrophages, in the LN and normal groups. Studies have reported that humoral and cellular immunity is involved in the pathogenesis of LN (29). A variety of autoantibodies that form immune complexes are deposited in the glomerulus, causing kidney tissue damage (30). Various immune cells can infiltrate kidney tissues. B-cell infiltration can produce many antibodies, causing kidney tissue damage and aggravating local inflammation. Activated T cells infiltrate kidney tissue and secrete cytokines, causing kidney damage. Macrophages activate a variety of signaling pathways and promote inflammation (31). They can cause glomerular mesangial matrix proliferation, innate cells, and damage to the structure or function of the kidney tissue (32). Macrophages can also release a large number of chemical and inflammatory mediators that aggravate kidney damage (33).

In this study, we found that S100A8 was differentially regulated in the above two microarray datasets between LN and normal glomeruli. Using immunofluorescence staining, we found that S100A8 levels in the controls were weak. Glomerular staining was markedly enhanced compared to that in the controls, especially in class IV. Protein S100A8 belongs to the calcium-binding S100 protein family and has gained considerable interest as a critical modulator of inflammatory response after its cellular release (34). Basic and clinical studies have suggested a potential link between S100A8 and LN (7, 8). Consistent with our research, Frosch et al. (35) reported that the expression pattern of S100A8 markedly differed between the glomeruli and interstitium in LN. S100A8 expression was significantly increased in the interstitium, paralleling the findings in glomeruli. Intrarenal S100A8 expression is increased in refractory patients with ISN/RPS class III/IV LN (36). Davies et al. (37) found that serum and urine S100A8 levels were elevated in patients with SLE, and the urine/serum ratios were elevated in patients with active LN. Tantivitayakul et al. (38) detected S100A8 in infiltrating cells of glomeruli and peritubular capillaries.

Macrophage infiltration is associated with the severity of the inflammatory response, and macrophages express a large amount of S100A8, which participates in the pathogenesis of LN. Staining of S100A8 in patients with classes II and V was similar to that of the controls and was enhanced in classes III, IV, III+V, and IV+V. Therefore, we speculate that the pathogenesis of S100A8 varies in different pathological types. Unfortunately, we did not find a relationship between S100A8 levels and clinical or laboratory data. However, the exact mechanisms of pathogenesis remain unclear. Further research is required to confirm the role of S100A8 in LN. One study reported that S100A8 could be a promising therapeutic target for myocardial ischemia–reperfusion injury (39). This is an important question that needs to be explored in future research.

## Conclusions

We used bioinformatics to determine the DEGs between the LN glomerulus and normal glomerulus. Immunofluorescence staining was used to identify the expression level of S100A8 in various ISN/RPS classes of LN. We found that the number of monocytes and activated NK cells were upregulated in the LN glomeruli, and the glomerular S100A8 level differed in different pathological types. Glomerular S100A8 staining was markedly increased in LN glomeruli compared to that in the controls, especially in class IV. Our results indicate that S100A8 participates in the pathogenesis of LN, and the precise mechanisms of this process need to be explored in our follow-up research.

## Data Availability Statement

The datasets presented in this study can be found in online repositories. The names of the repository/repositories and accession number(s) can be found in the article/**Supplementary Material**.

## Ethics Statement

The studies involving human participants were reviewed and approved by the ethics committee of Fujian Provincial Hospital. The patients/participants provided their written informed consent to participate in this study.

## Author Contributions

WQ collected and analyzed clinical data and drafted the article. PM did immunofluorescence staining. YQ and GF helped WQ to collect and interpret data for the work. CZ and WZ designed this topic and approved the final version of manuscript. LH revised the manuscript carefully.

## Funding

This work was supported by Natural Science Foundation of Fujian province (Grant No.2019J01184, 2021J05065) and joint funds for the innovation of science and technology of Fujian province (Grant No.2020Y9027).

## Conflict of Interest

The authors declare that the research was conducted in the absence of any commercial or financial relationships that could be construed as a potential conflict of interest.

## Publisher’s Note

All claims expressed in this article are solely those of the authors and do not necessarily represent those of their affiliated organizations, or those of the publisher, the editors and the reviewers. Any product that may be evaluated in this article, or claim that may be made by its manufacturer, is not guaranteed or endorsed by the publisher.
