# Supplementary figures and images for: Glomerular Expression of S100A8 in Lupus Nephritis: An Integrated Bioinformatics Analysis
Source: Front Immunol. 2022 Apr 27;13:843576. doi: 10.3389/fimmu.2022.843576 (PMC9092496; doi:10.3389/fimmu.2022.843576)

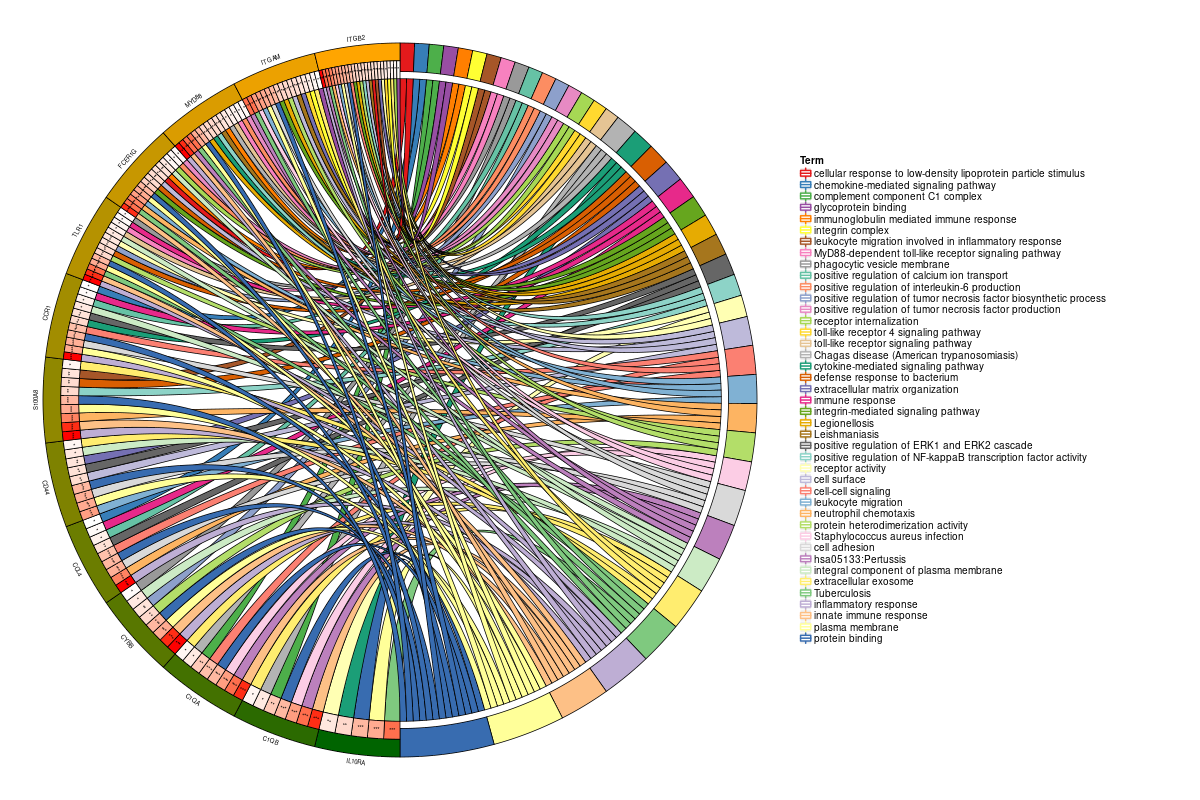

Supplement: Supplementary file 1 [file DataSheet_1.zip › test/circle_enrich.png]

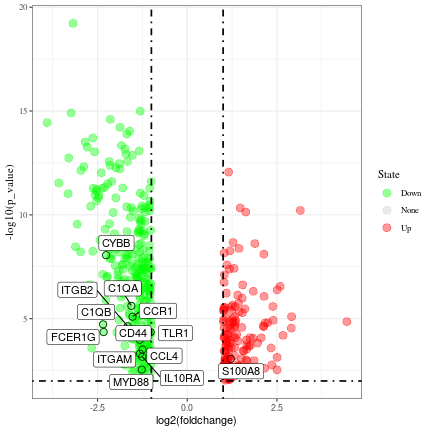

Supplement: Supplementary file 2 [file DataSheet_2.zip › volcano/volcano.png]

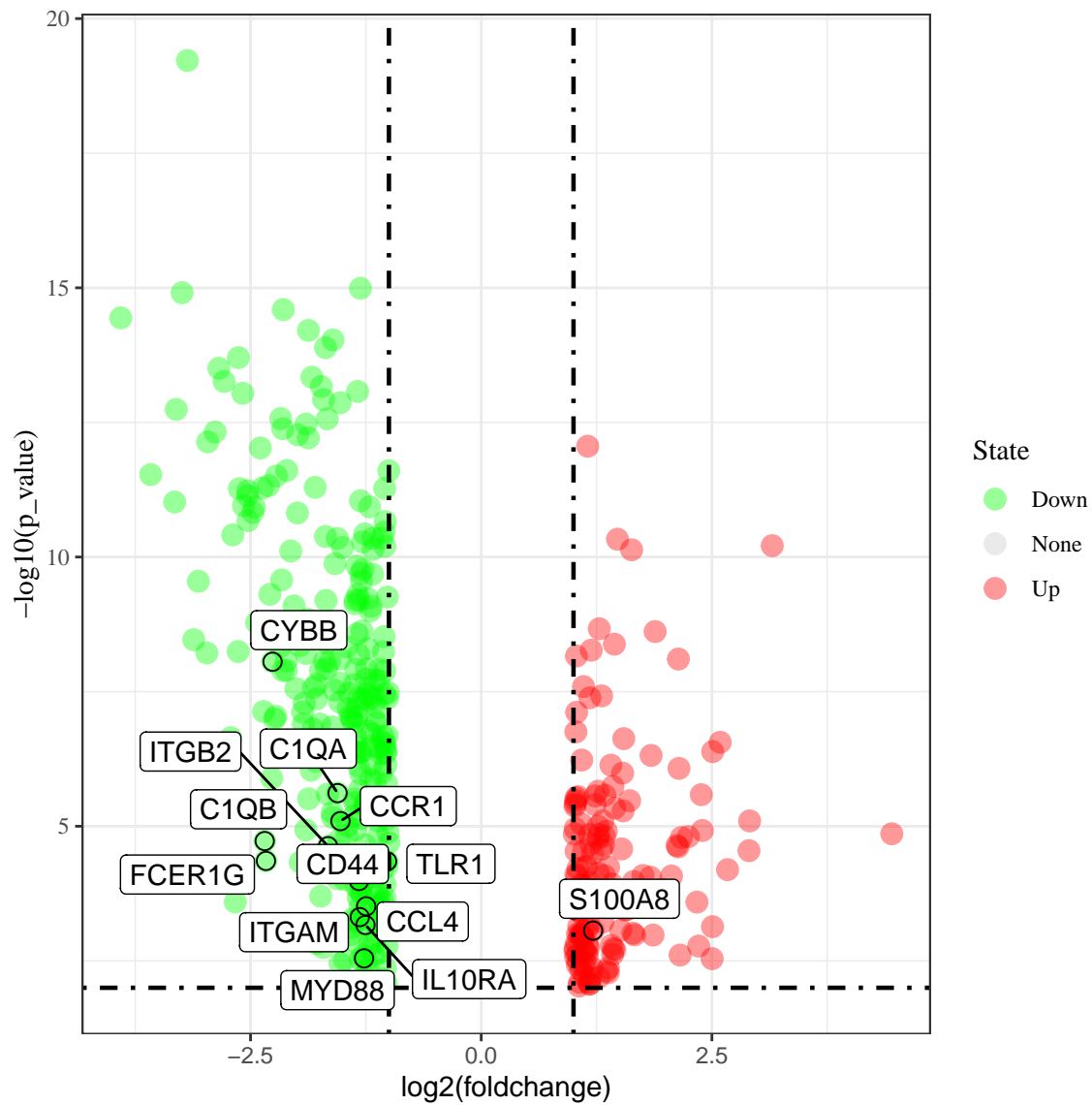

Supplement: Supplementary file 2 [file DataSheet_2.zip › volcano/volcano.pdf]

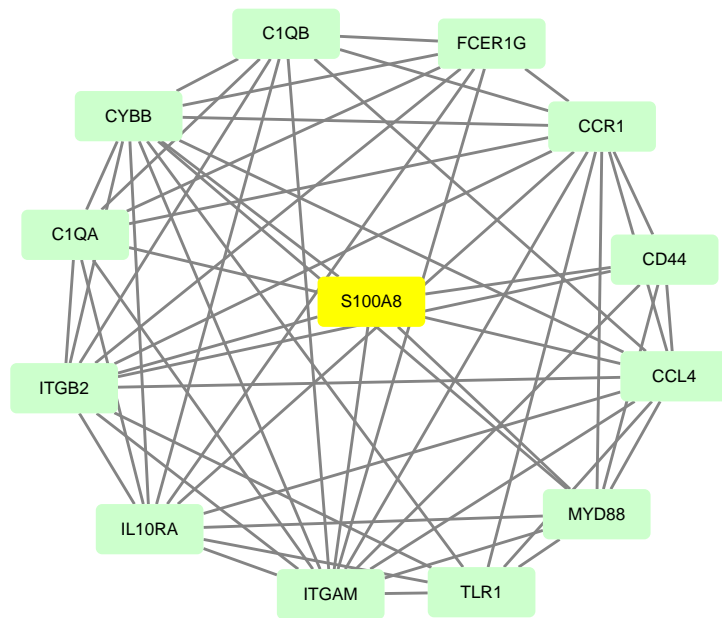

Supplement: Supplementary file 3 [file DataSheet_3.pdf]

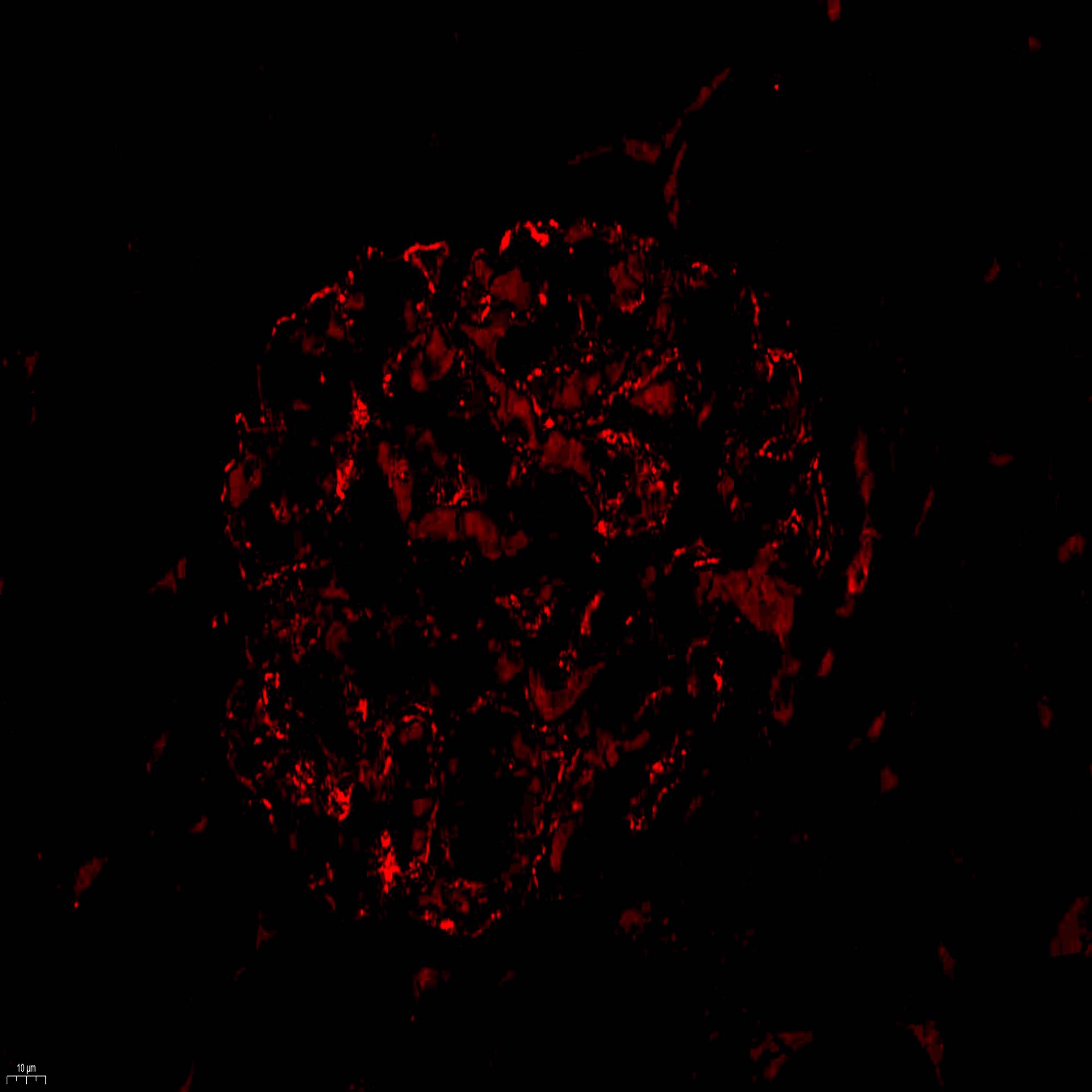

Supplement: Supplementary file 4 [file Image_1.jpeg]

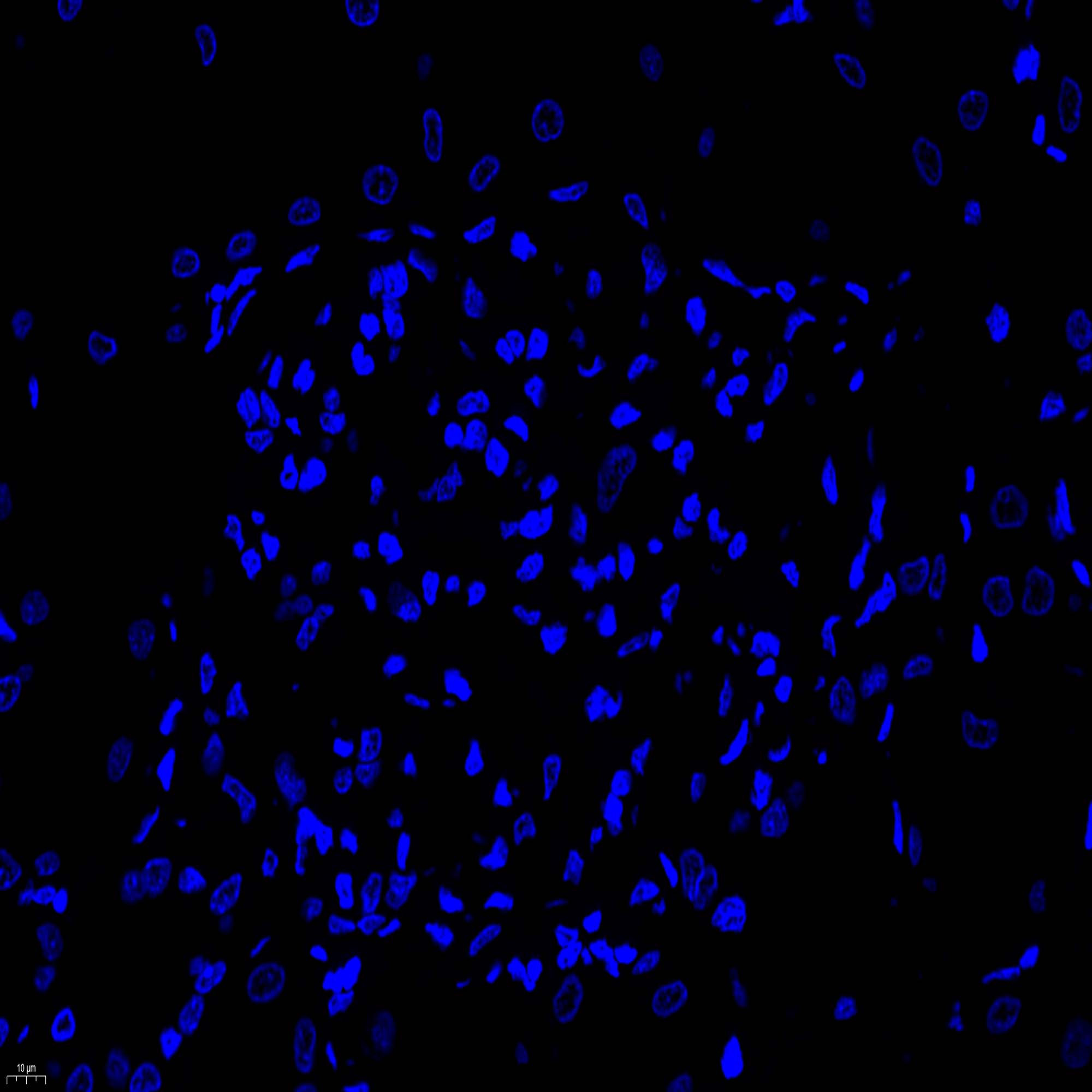

Supplement: Supplementary file 5 [file Image_2.jpeg]

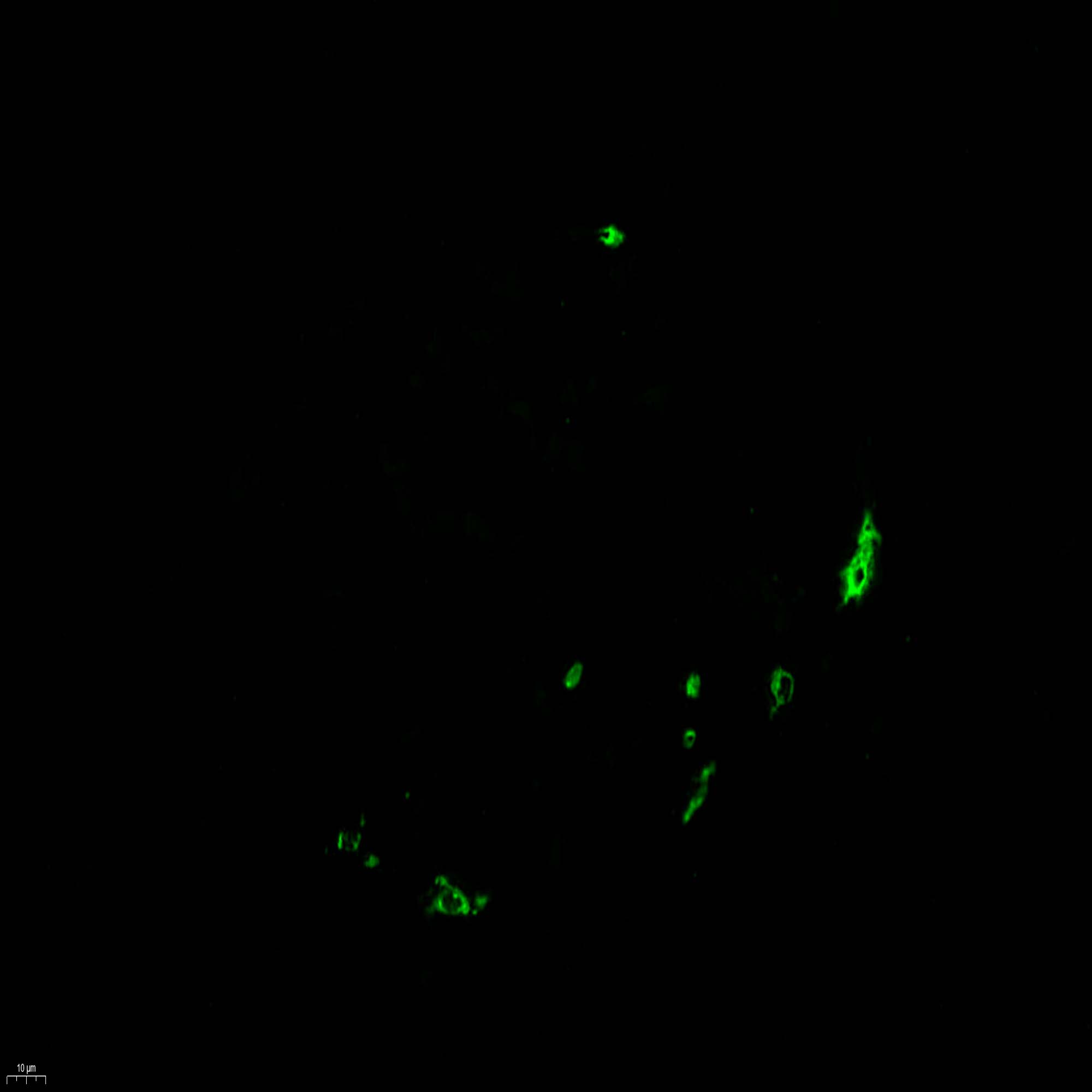

Supplement: Supplementary file 6 [file Image_3.jpeg]

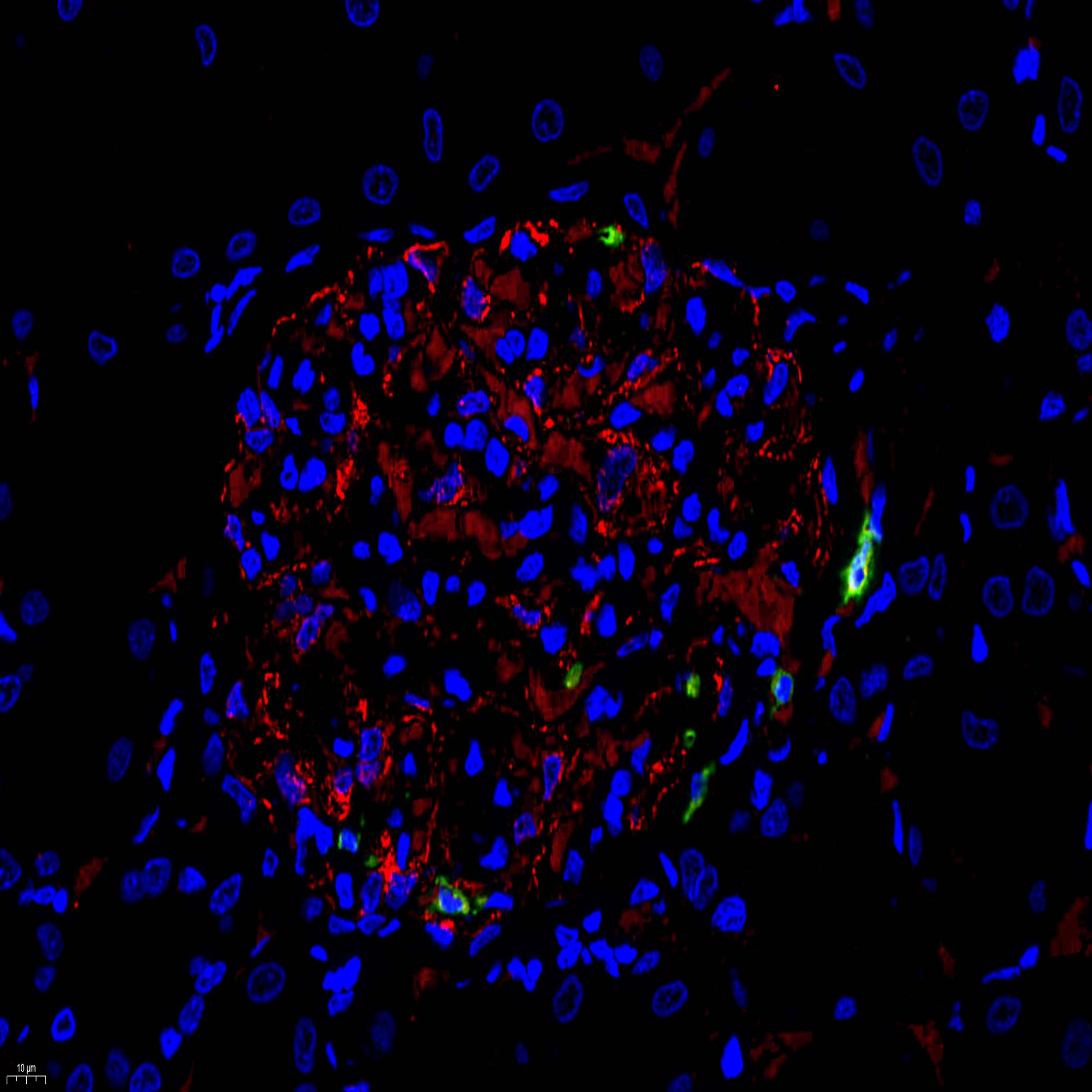

Supplement: Supplementary file 7 [file Image_4.jpeg]

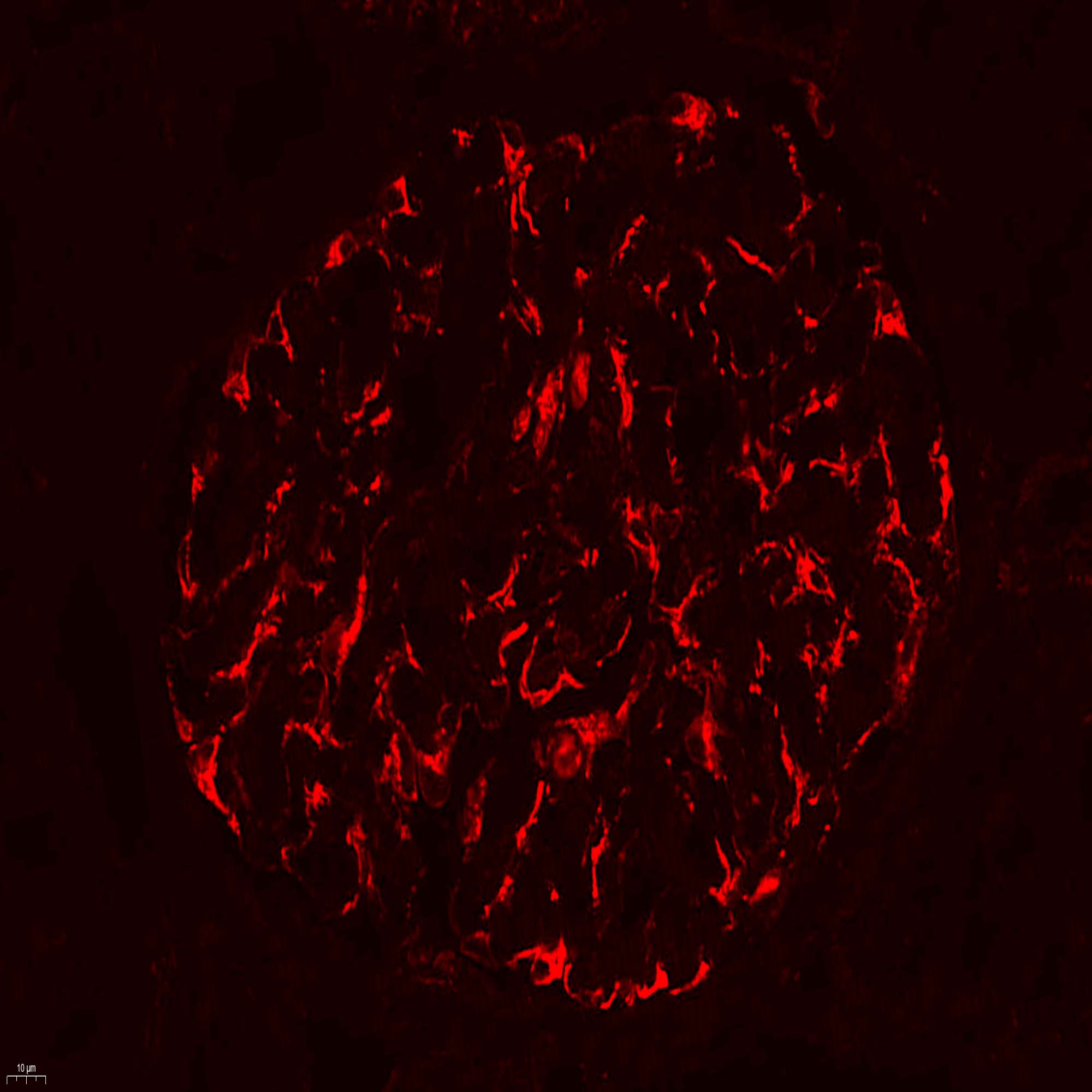

Supplement: Supplementary file 8 [file Image_5.jpeg]

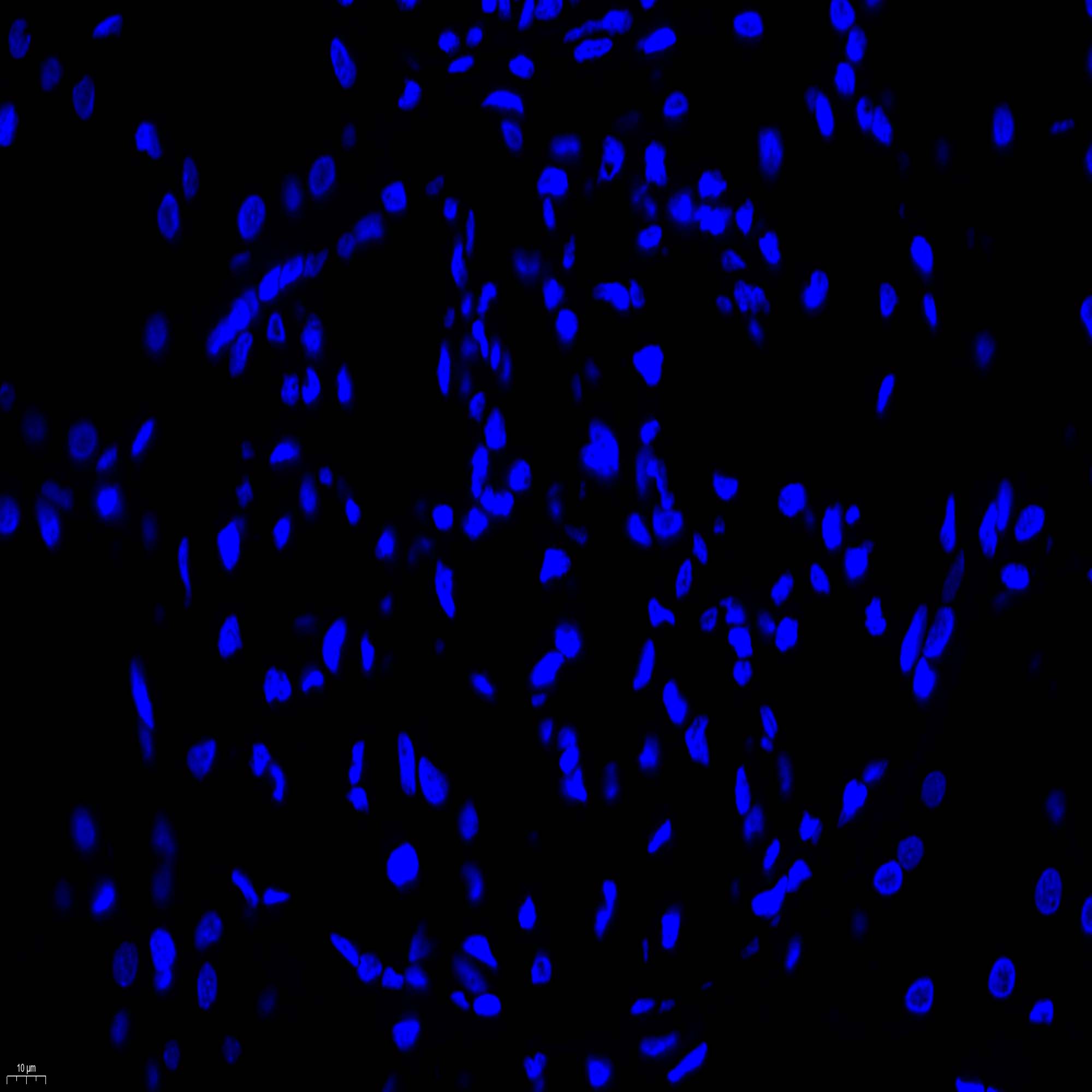

Supplement: Supplementary file 9 [file Image_6.jpeg]

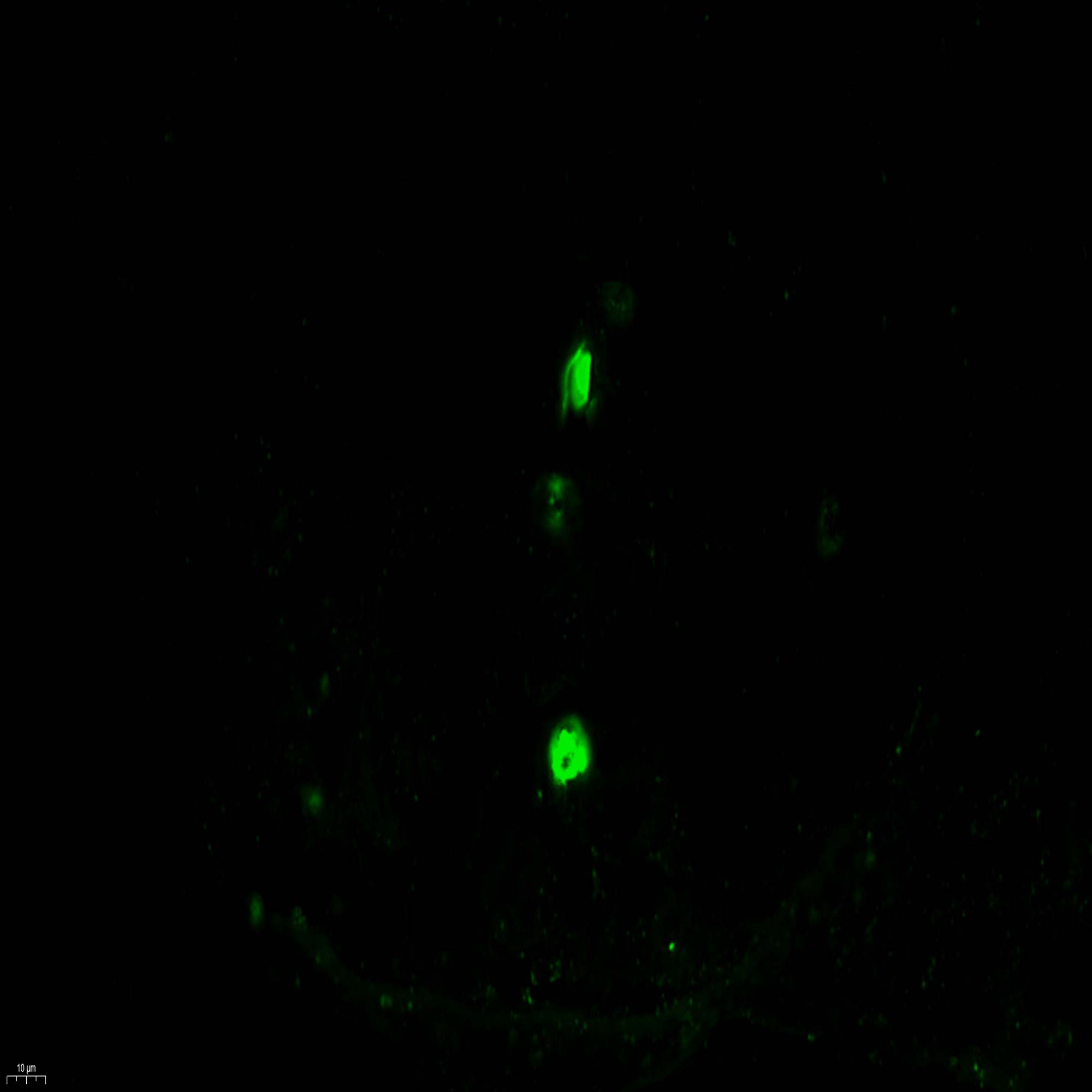

Supplement: Supplementary file 10 [file Image_7.jpeg]

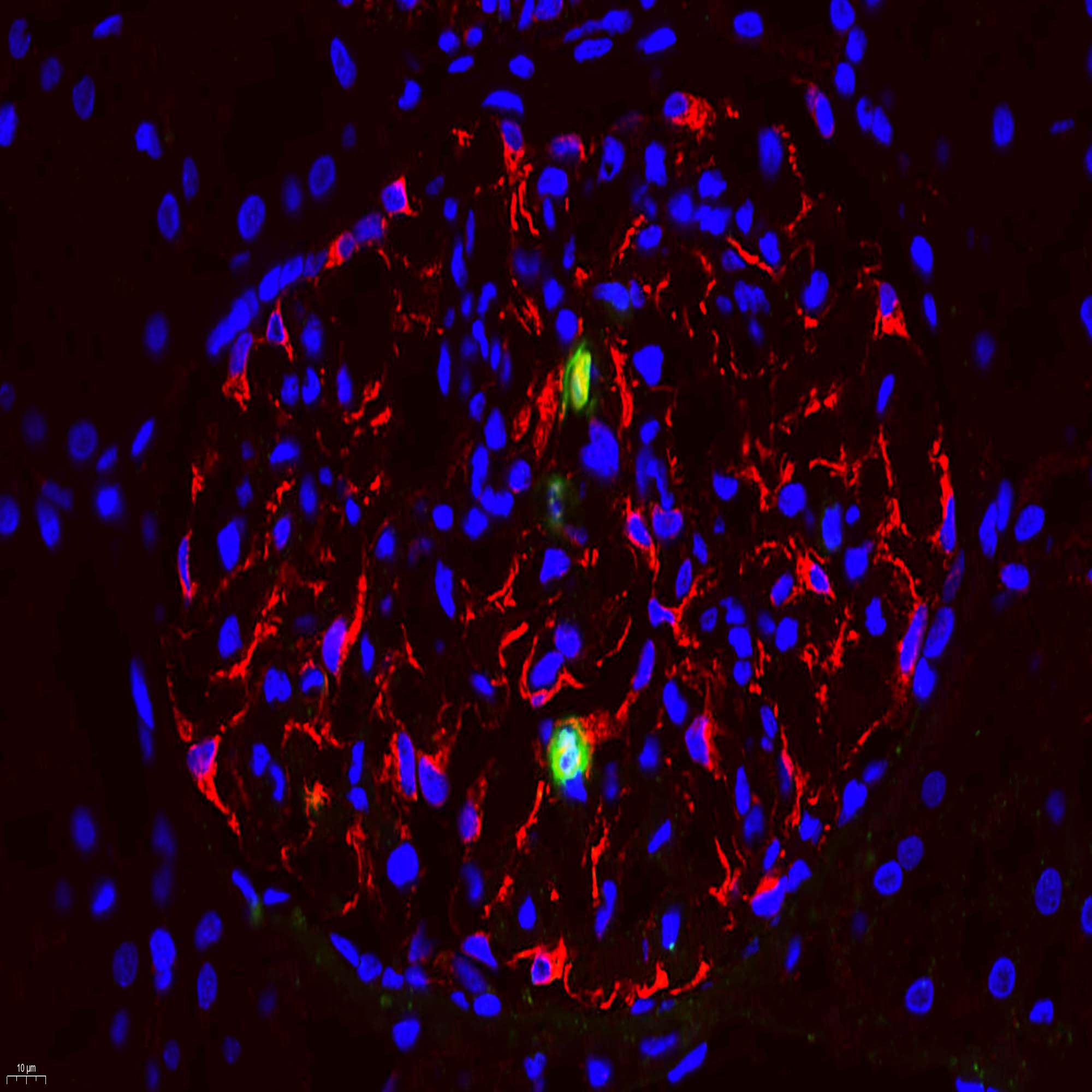

Supplement: Supplementary file 11 [file Image_8.jpeg]

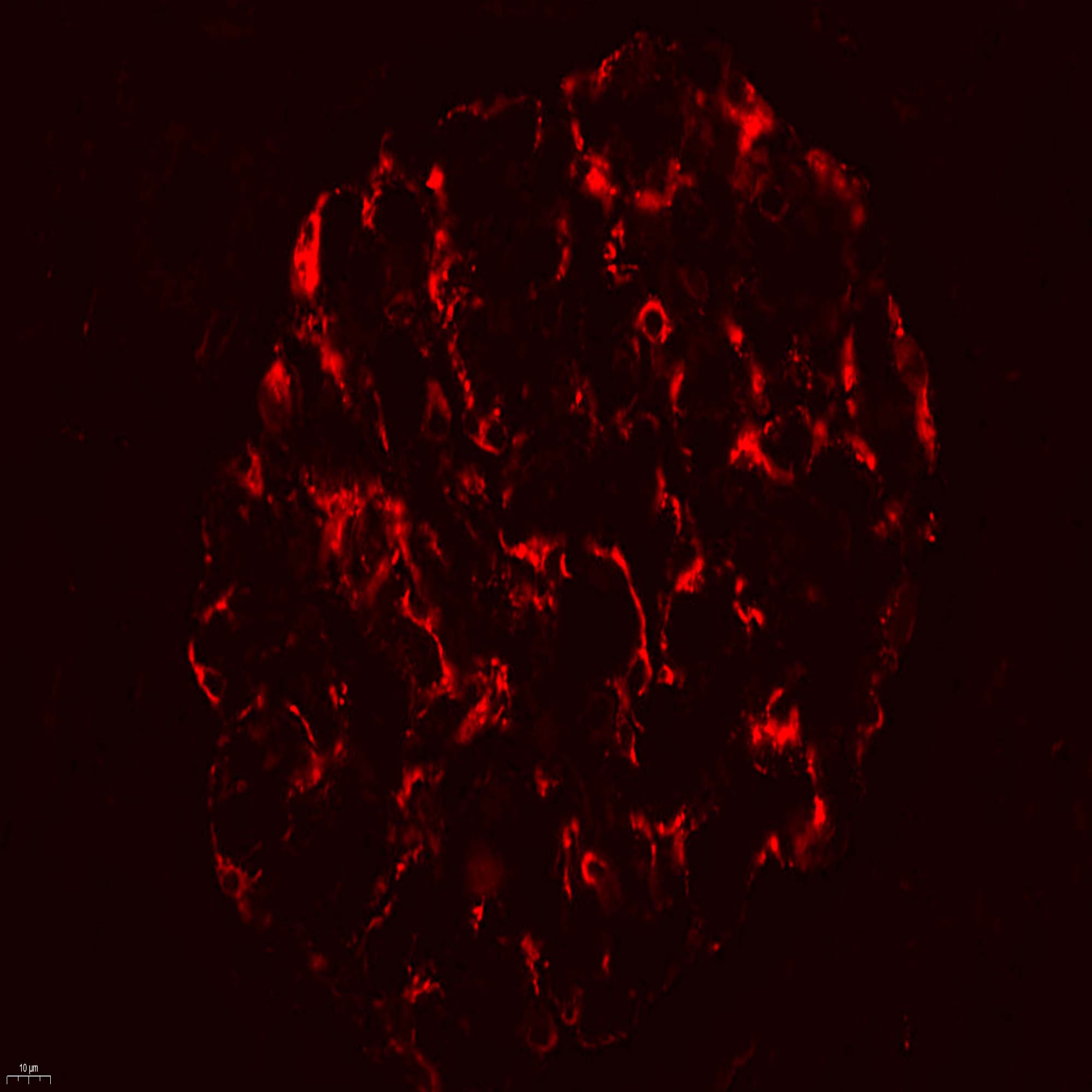

Supplement: Supplementary file 12 [file Image_9.jpeg]

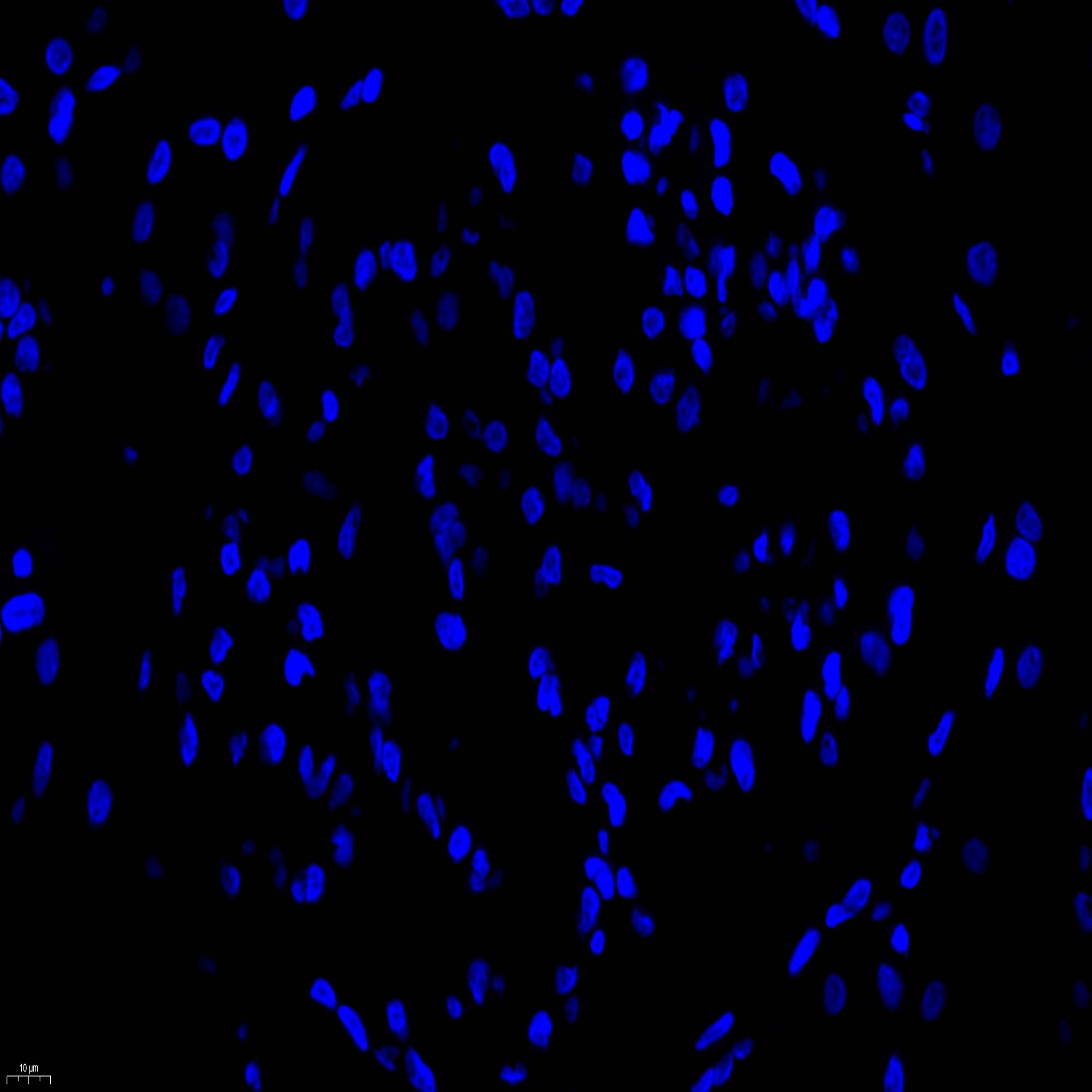

Supplement: Supplementary file 13 [file Image_10.jpeg]

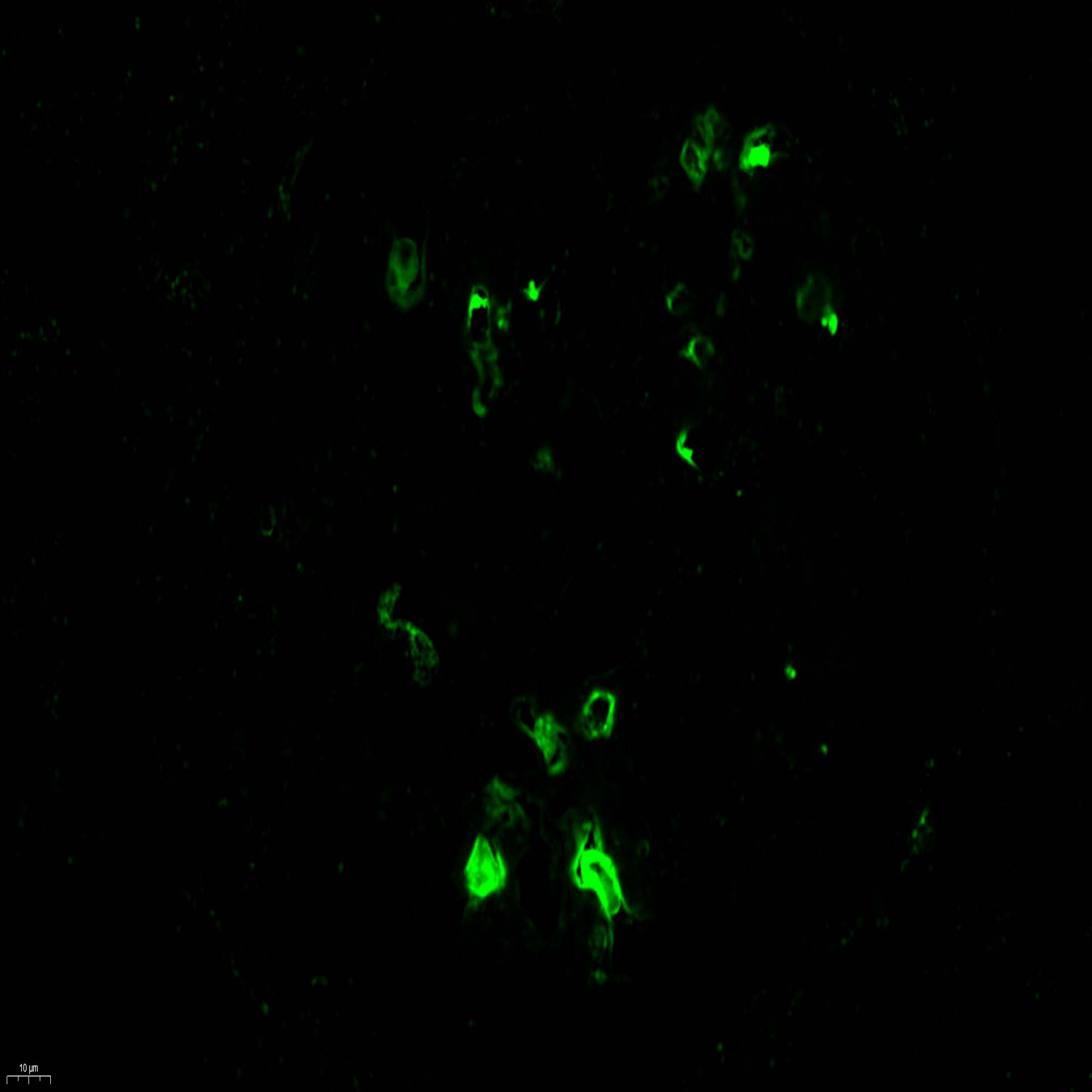

Supplement: Supplementary file 14 [file Image_11.jpeg]

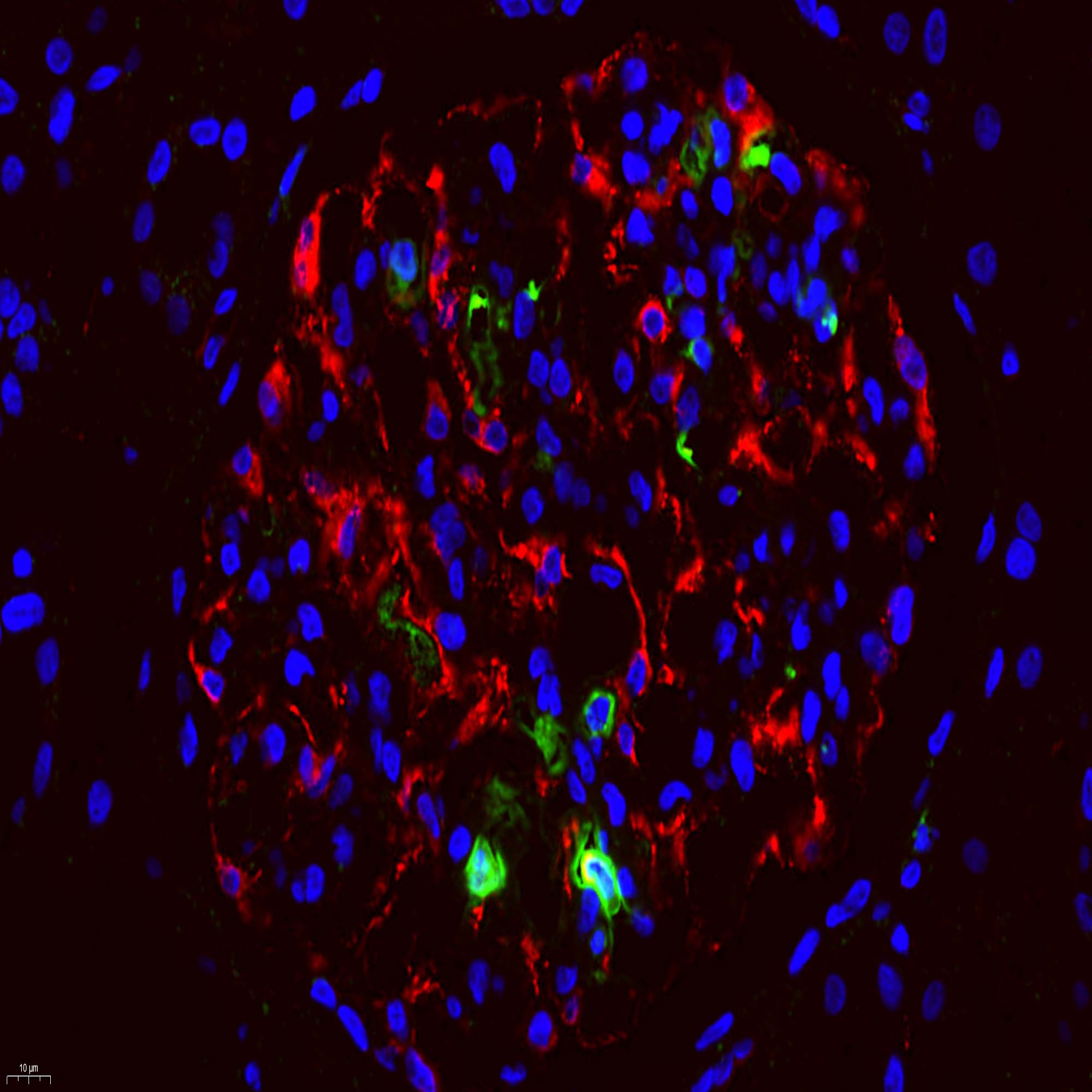

Supplement: Supplementary file 15 [file Image_12.jpeg]

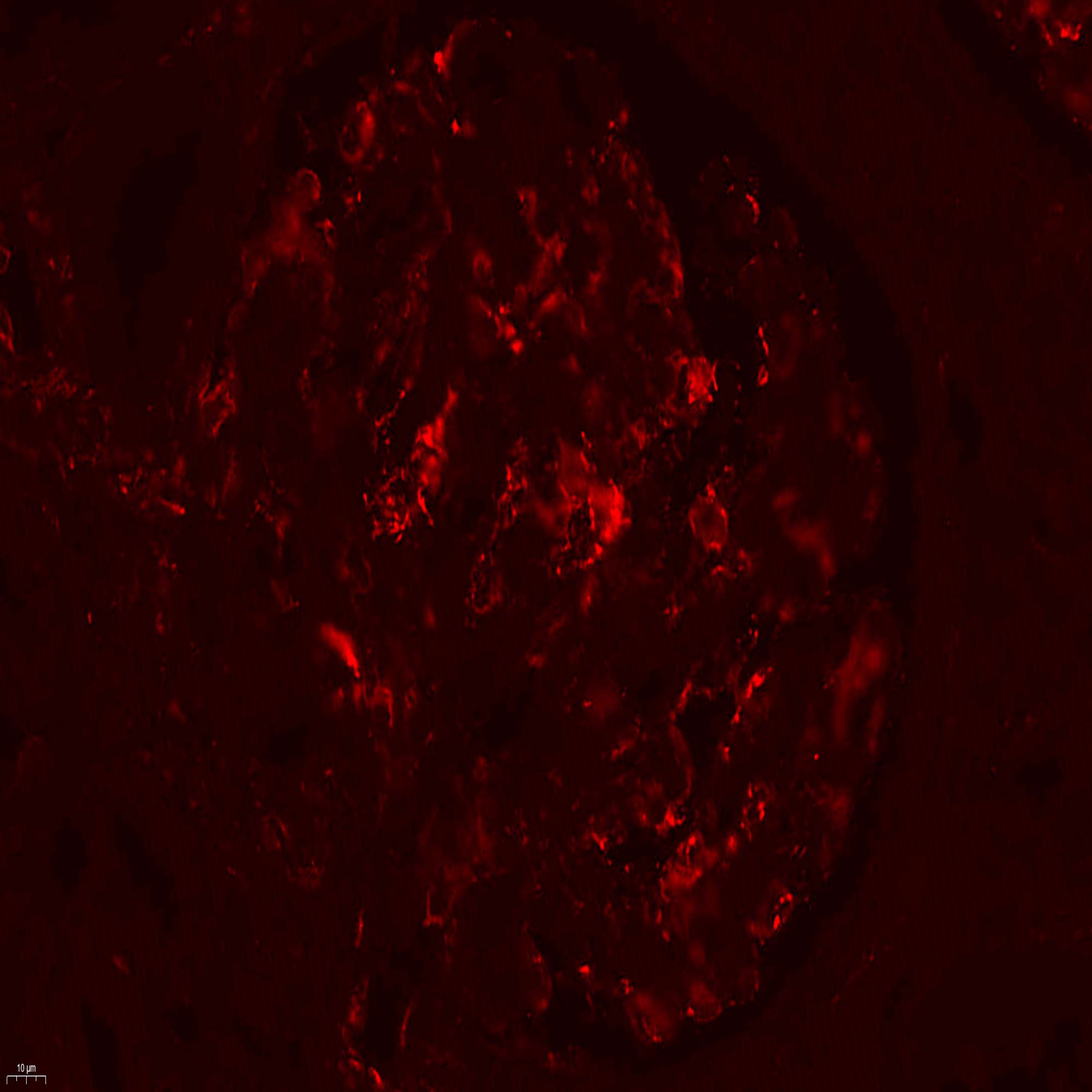

Supplement: Supplementary file 16 [file Image_13.jpeg]

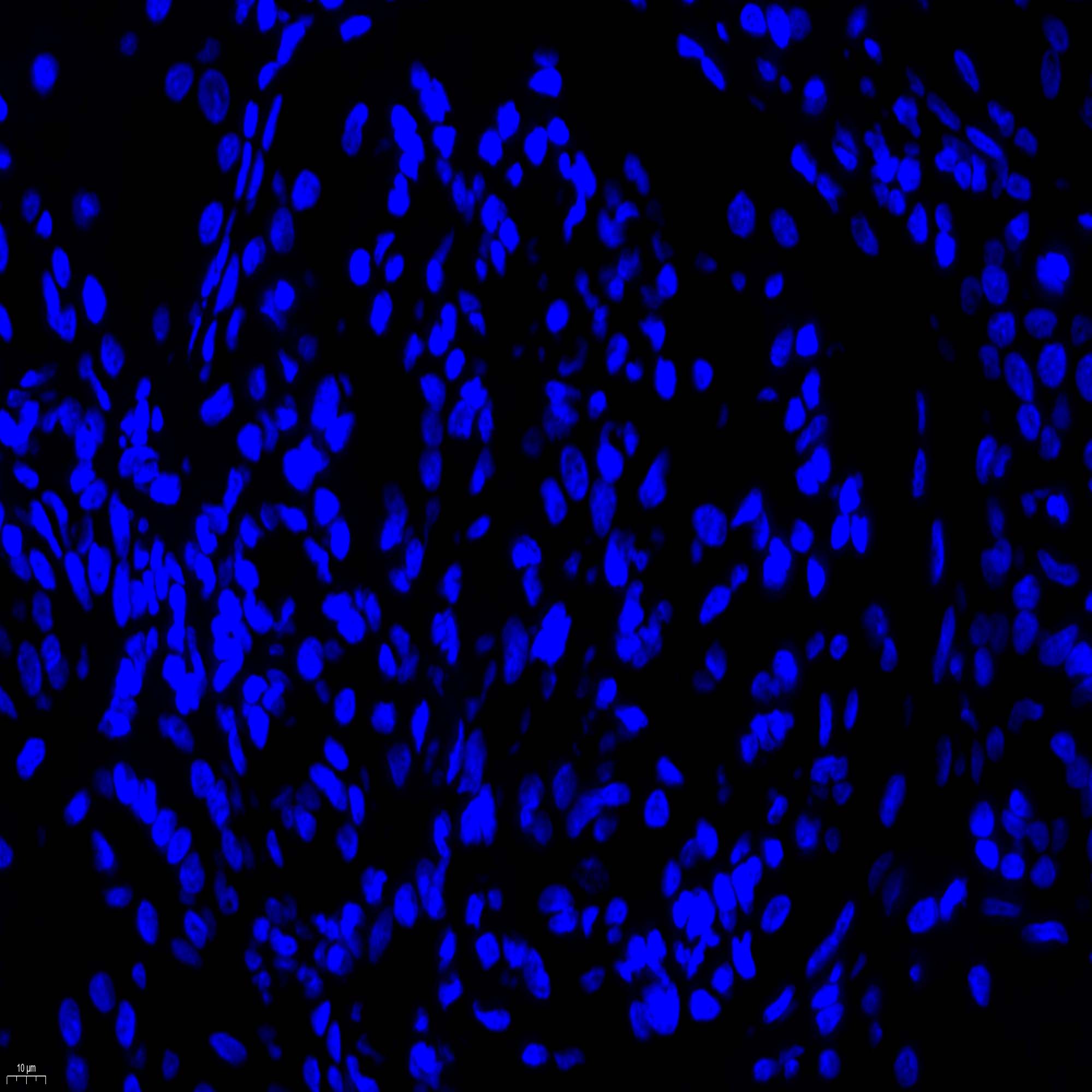

Supplement: Supplementary file 17 [file Image_14.jpeg]

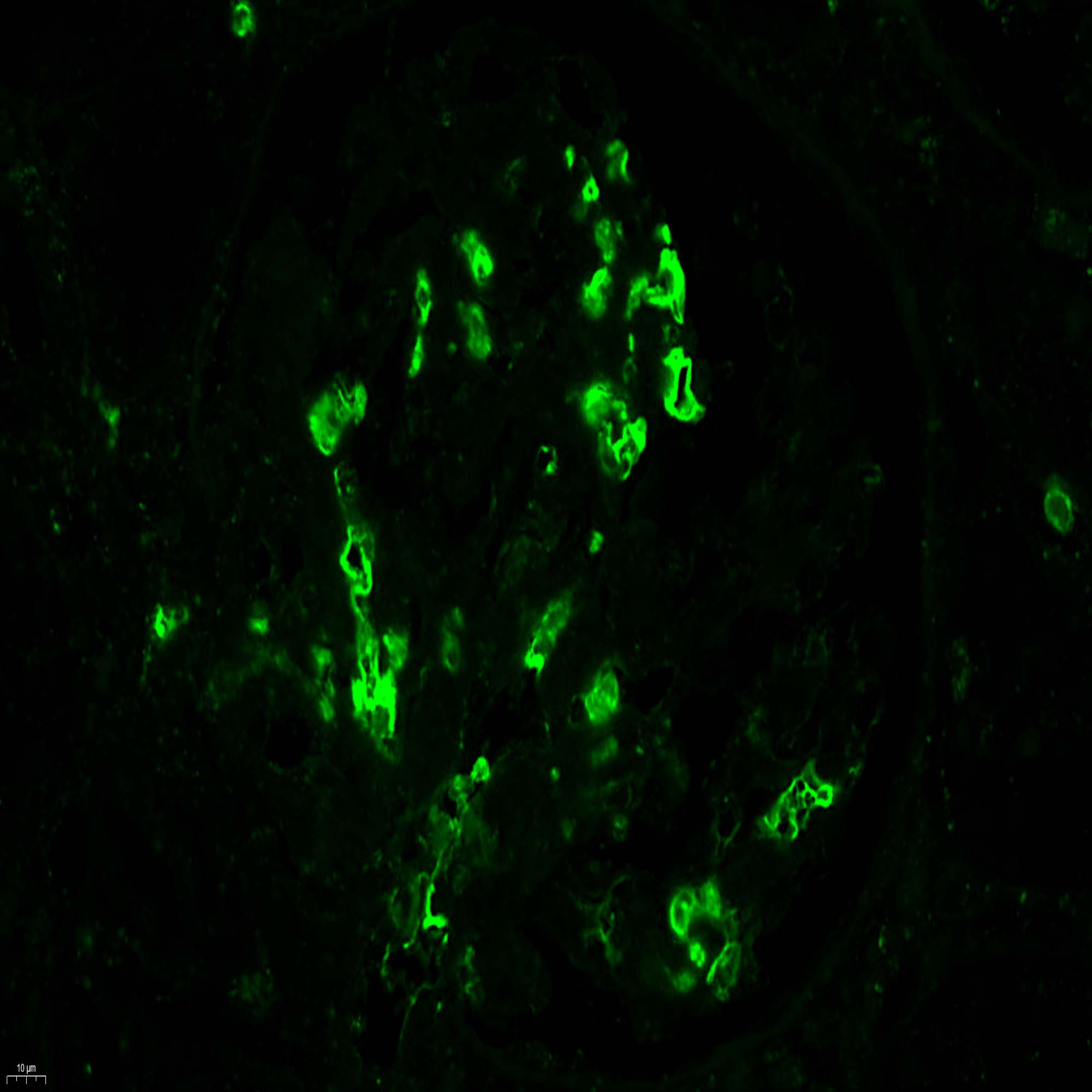

Supplement: Supplementary file 18 [file Image_15.jpeg]
