# Supplementary material for: Glomerular Expression of S100A8 in Lupus Nephritis: An Integrated Bioinformatics Analysis
Source: Front Immunol. 2022 Apr 27;13:843576. doi: 10.3389/fimmu.2022.843576 (PMC9092496; doi:10.3389/fimmu.2022.843576)
Supplement: Supplementary file 19 [file Table_1.docx]

**Table 1 GO and KEGG pathway enrichment analysis of DEGs in LN glomerulus.**

|  | Term | Description | Count in gene set | P-value |
| --- | --- | --- | --- | --- |
| KEGG_PATHWAY | hsa05133 | Pertussis | 5 | 4.06E-06 |
|  | hsa05152 | Tuberculosis | 6 | 4.34E-06 |
|  | hsa05150 | Staphylococcus aureus infection | 4 | 7.22E-05 |
|  | hsa05134 | Legionellosis | 3 | 0.003179 |
|  | hsa05140 | Leishmaniasis | 3 | 0.00544 |
|  | hsa05142 | Chagas disease (American trypanosomiasis) | 3 | 0.011392 |
|  | hsa04620 | Toll-like receptor signaling pathway | 3 | 0.011815 |
| biological processes (BP) | GO:0006954 | inflammatory response | 7 | 1.05E-07 |
|  | GO:0045087 | innate immune response | 7 | 2.21E-07 |
|  | GO:0030593 | neutrophil chemotaxis | 4 | 1.24E-05 |
|  | GO:0050900 | leukocyte migration | 4 | 7.85E-05 |
|  | GO:0007155 | cell adhesion | 5 | 2.29E-04 |
|  | GO:0007267 | cell-cell signaling | 4 | 6.80E-04 |
|  | GO:0007229 | integrin-mediated signaling pathway | 3 | 0.002185 |
|  | GO:0019221 | cytokine-mediated signaling pathway | 3 | 0.003787 |
|  | GO:0051092 | positive regulation of NF-kappaB transcription factor activity | 3 | 0.003901 |
|  | GO:0042742 | defense response to bacterium | 3 | 0.004618 |
|  | GO:0071404 | cellular response to low-density lipoprotein particle stimulus | 2 | 0.006415 |
|  | GO:0070374 | positive regulation of ERK1 and ERK2 cascade | 3 | 0.006655 |
|  | GO:0002523 | leukocyte migration involved in inflammatory response | 2 | 0.007835 |
|  | GO:0016064 | immunoglobulin mediated immune response | 2 | 0.007835 |
|  | GO:0042535 | positive regulation of tumor necrosis factor biosynthetic process | 2 | 0.007835 |
|  | GO:0030198 | extracellular matrix organization | 3 | 0.008283 |
|  | GO:0034142 | toll-like receptor 4 signaling pathway | 2 | 0.012792 |
|  | GO:0051928 | positive regulation of calcium ion transport | 2 | 0.018429 |
|  | GO:0002224 | toll-like receptor signaling pathway | 2 | 0.019131 |
|  | GO:0002755 | MyD88-dependent toll-like receptor signaling pathway | 2 | 0.023337 |
|  | GO:0031623 | receptor internalization | 2 | 0.03031 |
|  | GO:0032755 | positive regulation of interleukin-6 production | 2 | 0.031699 |
|  | GO:0032760 | positive regulation of tumor necrosis factor production | 2 | 0.033086 |
|  | GO:0006955 | immune response | 3 | 0.035053 |
|  | GO:0070098 | chemokine-mediated signaling pathway | 2 | 0.049591 |
| cell component (CC) | GO:0005886 | plasma membrane | 10 | 1.71E-04 |
|  | GO:0005602 | complement component C1 complex | 2 | 0.001317 |
|  | GO:0009986 | cell surface | 4 | 0.00471 |
|  | GO:0005887 | integral component of plasma membrane | 5 | 0.010799 |
|  | GO:0008305 | integrin complex | 2 | 0.01764 |
|  | GO:0070062 | extracellular exosome | 6 | 0.026716 |
|  | GO:0030670 | phagocytic vesicle membrane | 2 | 0.038177 |
| molecular function (MF) | GO:0005515 | protein binding | 13 | 3.93E-04 |
|  | GO:0046982 | protein heterodimerization activity | 4 | 0.003795 |
|  | GO:0004872 | receptor activity | 3 | 0.009973 |
|  | GO:0001948 | glycoprotein binding | 2 | 0.045254 |

GO, Gene Ontology; KEGG, Kyoto Encyclopedia of Genes and Genomes; DEGs, differentially expressed genes.
