# Supplementary material for: Glomerular Expression of S100A8 in Lupus Nephritis: An Integrated Bioinformatics Analysis
Source: Front Immunol. 2022 Apr 27;13:843576. doi: 10.3389/fimmu.2022.843576 (PMC9092496; doi:10.3389/fimmu.2022.843576)
Supplement: Supplementary file 20 [file Table_2.docx]

**Table 2 Clinical and laboratory information of the LN patients**

| Group | Control | LN patients | Class II | Class III | Class IV | Class V | Class III+V | Class IV+V | P value  (Control vs. LN) | P value  (Among LN) |
| --- | --- | --- | --- | --- | --- | --- | --- | --- | --- | --- |
| Age (years) | 55.47±8.82 | 32.70±12.17 | 31.60±11.89 | 36.9±17.61 | 31.00±12.75 | 35.40±11.15 | 33.40±12.08 | 30.80±11.97 | **＜0.01** | 0.05 |
| Sex (F/M) | 10/5 | 23/7 | 3/2 | 3/2 | 4/1 | 5/0 | 3/2 | 5/0 | 0.49 | 0.44 |
| SLEDAI | NA | 18.57±4.44 | 14.40±2.97 | 15.8±2.68 | 20.4±2.97 | 17.2±4.15 | 22.4±3.36 | 21.2±5.02 | NA | **0.009** |
| Cr (μmol/L) | 75.27±21.30 | 75.50±72.43 | 52.00±10.89 | 64.00±10.08 | 69.80±10.46 | 134.20±175.51 | 56.00±14.25 | 77.00±31.96 | 0.99 | **0.04** |
| BUN (mmol/L) | 4.89±0.86 | 7.00±4.83 | 5.38±1.53 | 7.20±1.60 | 4.92±0.64 | 8.70±8.47 | 3.88±0.90 | 11.90±6.04 | **0.03** | **＜0.01** |
| 24UTP or Urine protein (g/24h) | All negative | 1.66±1.71 | 0.95±1.10 | 0.70±0.47 | 1.53±0.65 | 2.18±1.57 | 2.16±1.75 | 2.40±3.25 | **＜0.01** | 0.54 |
| C3 (g/L) | NA | 0.52±0.09 | 0.53±0.24 | 0.68±0.37 | 0.29±0.14 | 0.89±0.35 | 0.36±0.16 | 0.37±0.09 | NA | **0.007** |
| C4 (g/L) | NA | 0.09±0.07 | 0.08±0.04 | 0.11±0.08 | 0.07±0.05 | 0.18±0.13 | 0.05±0.03 | 0.06±0.03 | NA | **0.06** |
| Alb (g/L) | NA | 32.33±6.58 | 38.20±4.15 | 33.20±7.40 | 26.60±7.40 | 35.80±3.70 | 31.60±2.61 | 28.60±6.95 | NA | **0.04** |
