# Supplementary material for: Glomerular Expression of S100A8 in Lupus Nephritis: An Integrated Bioinformatics Analysis
Source: Front Immunol. 2022 Apr 27;13:843576. doi: 10.3389/fimmu.2022.843576 (PMC9092496; doi:10.3389/fimmu.2022.843576)
Supplement: Supplementary file 21 [file Table_3.docx]

| **Glomerular S100A8-AR/IOD**  **P value** | **Control** | **LN patients** | **Class II** | **Class III** | **Class IV** | **Class V** | **Class III+V** | **Class IV+V** |
| --- | --- | --- | --- | --- | --- | --- | --- | --- |
| **Control** | **0.002 (0.001, 0.005)/**  **5.967(2.149, 16.933)** | **0.002/**  **0.006** | 0.955/  0.966 | **＜0.001/**  **0.001** | **＜0.001/**  **＜0.001** | 0.856/  0.916 | **0.002/**  **0.013** | **＜0.001/**  **＜0.001** |
| **LN patients** | **0.002/**  **0.006** | **0.010 (0.002, 0.028)/**  **24.805 (5.647, 87.4068)** | **＜0.001/**  **＜0.001** | **0.003**/  0.387 | **＜0.001/**  **＜0.001** | **0.001/**  **0.005** | 0.988/  0.693 | 0.601/  0.312 |
| **Class II** | 0.955/  0.966 | **＜0.001/**  **＜0.001** | **0.002 (0.001, 0.005)/**  **6.982 (2.161, 13.752)** | **＜0.001/**  **0.001** | **＜0.001/**  **＜0.001** | 0.814/  0.884 | **＜0.001/**  **0.015** | **＜0.001/**  **＜0.001** |
| **Class III** | **＜0.001/**  **0.001** | **0.003**/  0.387 | **＜0.001/**  **0.001** | **0.026 (0.020, 0.075)/**  **82.603 (20.480, 147.444)** | **＜0.001/**  **＜0.001** | **＜0.001/**  **0.001** | **＜0.001**/  0.218 | **＜0.001**/  0.887 |
| **Class IV** | **＜0.001/**  **＜0.001** | **＜0.001/**  **＜0.001** | **＜0.001/**  **＜0.001** | **＜0.001/**  **＜0.001** | **0.059 (0.035, 0.107)/**  **227.417 (133.910, 407.012)** | **＜0.001/**  **＜0.001** | **＜0.001/**  **＜0.001** | **＜0.001/**  **＜0.001** |
| **Class V** | 0.856/  0.916 | **0.001/**  **0.005** | 0.814/  0.884 | **＜0.001/**  **0.001** | **＜0.001/**  **＜0.001** | **0.003 (0.000, 0.003)/**  **1.027 (0.000, 10.880)** | **＜0.001/**  **0.016** | **＜0.001/**  **＜0.001** |
| **Class III+V** | **0.002/**  **0.013** | 0.988/  0.693 | **＜0.001/**  **0.015** | **＜0.001**/  0.218 | **＜0.001/**  **＜0.001** | **＜0.001/**  **0.016** | **0.013 (0.006, 0.033)/**  **39.839 (14.546, 90.071)** | 0.632/0.208 |
| **Class IV+V** | **＜0.001/**  **＜0.001** | 0.601/  0.312 | **＜0.001/**  **＜0.001** | **＜0.001**/  0.887 | **＜0.001/**  **＜0.001** | **＜0.001/**  **＜0.001** | 0.632/0.208 | **0.018 (0.008, 0.034)/**  **62.562 (20.069, 100.833)** |

**Table 3 Glomerular expression of S100A8 in various ISN/RPS class LN patients**
