# Supplementary material for: Glomerular Expression of S100A8 in Lupus Nephritis: An Integrated Bioinformatics Analysis
Source: Front Immunol. 2022 Apr 27;13:843576. doi: 10.3389/fimmu.2022.843576 (PMC9092496; doi:10.3389/fimmu.2022.843576)
Supplement: Supplementary file 1 [file circle_enrich.pdf › test/circle_enrich.pdf]

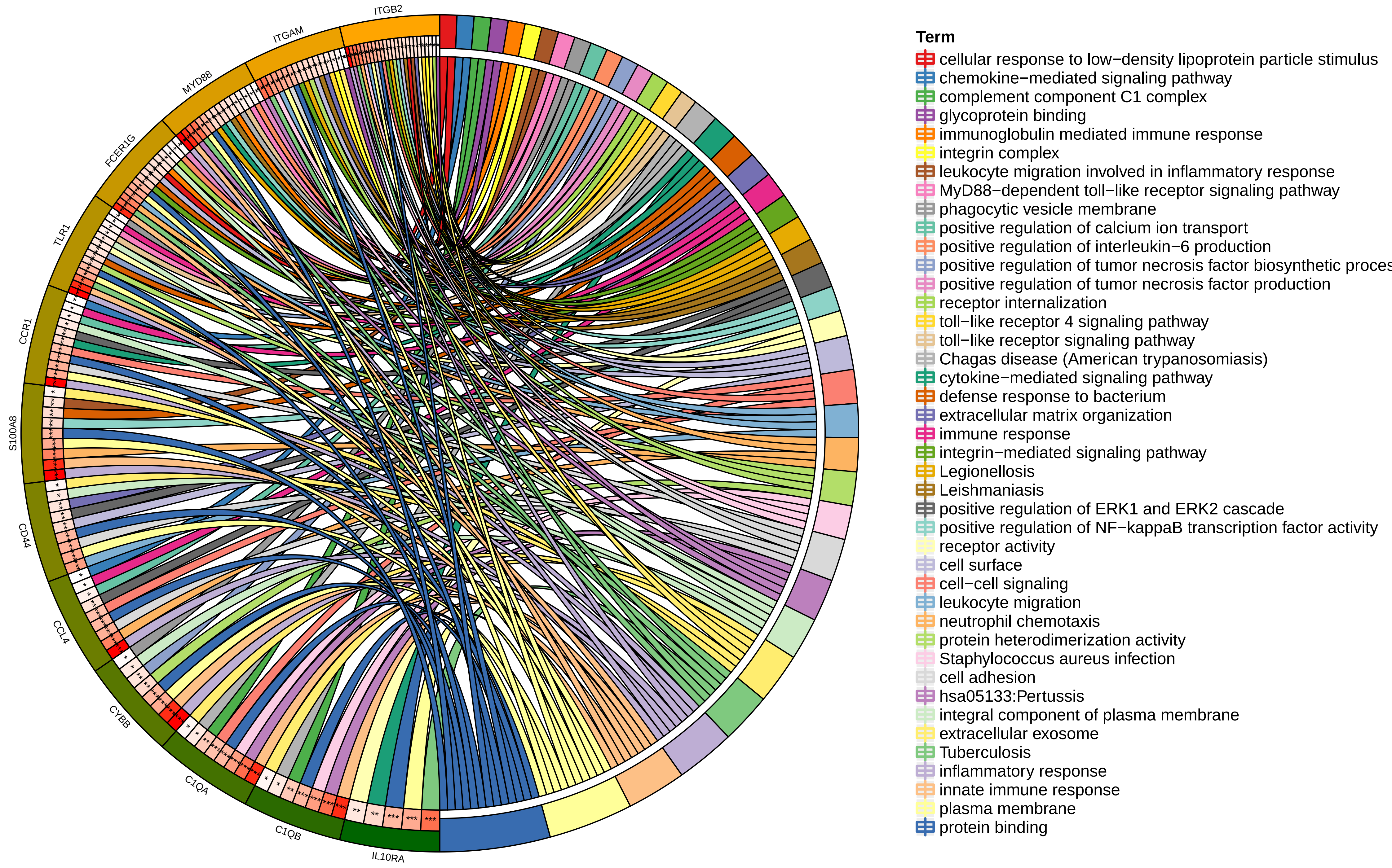
ITGB2
ITGAM
MYD88
FCER1G
TLR1
CCR1
S100A8
CD44
CCL4
CYBB
C1QA
C1QB
IL10RA
Term
cellular response to low−density lipoprotein particle stimulus
chemokine−mediated signaling pathway
complement component C1 complex
glycoprotein binding
immunoglobulin mediated immune response
integrin complex
leukocyte migration involved in inflammatory response
MyD88−dependent toll−like receptor signaling pathway
phagocytic vesicle membrane
positive regulation of calcium ion transport
positive regulation of interleukin−6 production
positive regulation of tumor necrosis factor biosynthetic proces
positive regulation of tumor necrosis factor production
receptor internalization
toll−like receptor 4 signaling pathway
toll−like receptor signaling pathway
Chagas disease (American trypanosomiasis)
cytokine−mediated signaling pathway
defense response to bacterium
extracellular matrix organization
immune response
integrin−mediated signaling pathway
Legionellosis
Leishmaniasis
positive regulation of ERK1 and ERK2 cascade
positive regulation of NF−kappaB transcription factor activity
receptor activity
cell surface
cell−cell signaling
leukocyte migration
neutrophil chemotaxis
protein heterodimerization activity
Staphylococcus aureus infection
cell adhesion
hsa05133:Pertussis
integral component of plasma membrane
extracellular exosome
Tuberculosis
inflammatory response
innate immune response
plasma membrane
protein binding
